# Fatal cases involving new psychoactive substances and trends in analytical techniques

**DOI:** 10.3389/ftox.2022.1033733

**Published:** 2022-10-25

**Authors:** Ettore Ferrari Júnior, Bruno Henrique Monteiro Leite, Eliude Barbosa Gomes, Tales Mateus Vieira, Pedro Sepulveda, Eloisa Dutra Caldas

**Affiliations:** ^1^ Forensic Analysis Laboratory, Criminalistics Institute, The Civil Police of the Federal District, Brasília, Brazil; ^2^ Laboratory of Toxicology, Department of Pharmacy, University of Brasília, Campus Darcy Ribeiro, Brasília, Brazil; ^3^ Brainfarma Pharmaceutical Company, Anápolis, Brazil; ^4^ Department of Pharmacy, University of Brasília, Campus Ceilândia, Brasília, Brazil

**Keywords:** new psychoactive substances, GC-MS, LC-MS/MS, HRMS, opioids, synthetic cathinones, fatal cases

## Abstract

New psychoactive substances (NPS) are an emerging public health issue and deaths are commonly associated with polydrug abuse. Moreover, the number of new substances available is constantly increasing, causing intoxications in low doses, characteristics that impose to toxicology and forensic laboratories to keep routine methods up to date, with high detectability and constantly acquiring new analytical standards. Likewise, NPS metabolites and respective elimination pathways are usually unknown, making it difficult the detection and confirmation of the drug involved in the fatal case in an analytical routine. A literature search was performed on PubMed, Scopus and Web of Science databases for papers related to chromatographic analyses from fatal cases related to NPS use published from 2016 to 2021. A total of 96 papers were retrieved and reviewed in this study. Opioids, synthetic cathinones, phenethylamines/amphetamines and cannabinoids were the NPS classes most found in the fatal cases. In many cases, multiple compounds were detected in the biological samples, including prescription and other illegal drugs. Liquid chromatography-tandem mass spectrometry, an alternative to overcome the gas chromatography–mass spectrometry limitations for some compounds, was the analytical technique most used in the studies, and high resolution mass spectrometry was often applied to NPS metabolite investigation and structural characterization and identification of unknown compounds. Toxicological screening and quantitation methods need to be continuously updated to include new substances that are emerging on the drug market that can be fatal at very low doses.

## Introduction

New psychoactive substances (NPS) are drugs that are not scheduled under the 1961 United Nations Single Convention on Narcotic Drugs or the 1971 United Nations Convention on Psychotropic Substances, and are synthetized to mimic the effect of traditional drugs (UNODC, 2021a). The illicit market of NPS has been constantly changing due to introduction of new substances, which brings potential new public health problems, since little is known about their toxicology, with reporting of fatal poisoning cases worldwide (UNODC, 2020).

**FIGURE 1:**
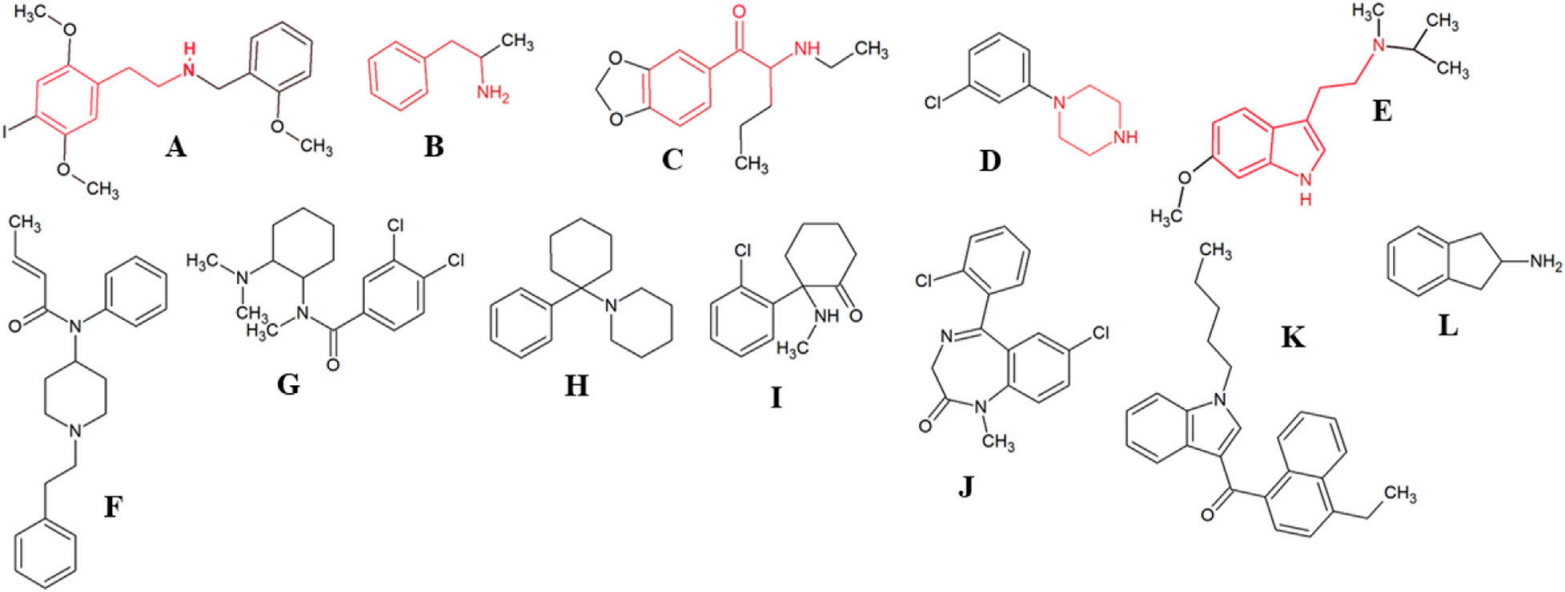
Chemical structure of the main compounds from the NPS classes discussed in this paper. 25I-NBOMe **(A)** and amphetamine **(B)**, highlighting the phenethylamine core (red); the synthetic cathinone N-ethylpentylone **(C)**, highlighting the cathinone core (red); m-CPP **(D)**, highlighting the piperazine core (red); 5-MeO-MIPT **(E)**, highlighting the tryptamine core (red); Opioids: fentanyl analog crotonylfentanyl **(F)** and U-47700 **(G)**; phencyclidine **(H)** and the analogue ketamine **(I)**; designer benzodiazepine diclazepam **(J)**; synthetic cannabinoid JWH-210 **(K)**; 2-Aminoindane **(L)**.

Some NPS are classified as a group based on structural similarity and/or psychoactive effects. Furthermore, there are also similarities among the NPS groups, as example, phenethylamines also include amphetamines, which have structures similar to cathinones. With the same quickness that the NPS appear in the market, they are replaced for other analogs to escape from the official control of illegal substances (EMCDDA, 2021a), which brings a constant challenge for forensic laboratories that uses mostly chromatographic techniques to elucidate intoxication cases. Figure 1 shows the chemical structure of the main NPS groups discussed in this review.

Gas chromatography coupled with mass spectrometer detector (GC-MS) is a robust analytical instrumentation applied to systematic toxicological analysis, which is available in most forensic laboratories, providing unequivocal molecular identification and acceptable limits of detection for the majority of compounds of toxicological interest (Rojkiewicz et al., 2016; Ellefsen et al., 2017; Atherton et al., 2018; Dwyer et al., 2018; Majchrzak et al., 2018; Ivanov et al., 2019; Tiemensma et al., 2020; Woods, 2020; Cartiser et al., 2021). However, liquid chromatography-tandem mass spectrometry (LC-MS/MS) methods are able to overcome analytical limitations of the GC techniques, such as thermal degradation (Ferrari Júnior et al., 2020), providing lower detection limits that are needed for some compounds, as synthetic cannabinoids (Gieron and Adamowicz, 2016; Shanks and Behonick, 2016; Angerer et al., 2017; Adamowicz et al., 2019; Zawadski et al., 2020a; Hvozdovich et al., 2020; Krotulski et al., 2021a), opioids (Guerrieri et al., 2017; Krotulski et al., 2017; Krotulski et al., 2021b, 2021c; Castelino et al., 2021; Mueller et al., 2021), and phenethylamines (Kristofic et al., 2016). In some cases, the suspicion of intoxication may involve an unknown substance for the laboratory routine, a problem that can be solved using high resolution mass spectrometry (HRMS), that features high mass accuracy as a tool for untargeted screening analysis (Deville et al., 2019; Fels et al., 2019; Gaulier et al., 2019; Kovacs et al., 2019; Nash et al., 2019; Theofel et al., 2019; Yeter and Erol Öztürk, 2019; Krotulski et al., 2021d). HRMS techniques can be also applied to NPS metabolite investigation, which can be essential to confirm the use of the drugs, mainly those that are rapidly metabolized (Allibe et al., 2018; Krotulski et al., 2020a). Furthermore, the analysis of seized drugs and other materials found near the victim can bring additional information that helps to elucidate the intoxication case (Rojkiewicz et al., 2016; Strehmel et al., 2018).

The non-detection and underreporting of NPS in postmortem analysis and the absence of toxicological studies to establish possible risks caused by NPS consumption make difficult to understand the real impact of NPS in fatal intoxication cases (EMCDDA, 2021a). Although there are some reviews on analytical techniques for NPS detection, a review that covers both the toxicological aspects of acute fatal cases and the analytical strategies used in postmortem analysis is limited in the literature.

The aim of the present paper was to review the literature published from 2016 to 2021 concerning fatal cases that involved NPS abuse and the analytical methods applied in toxicological analyses, to understand how laboratories have been dealing with those emerging drugs.

## Method

**FIGURE 2:**
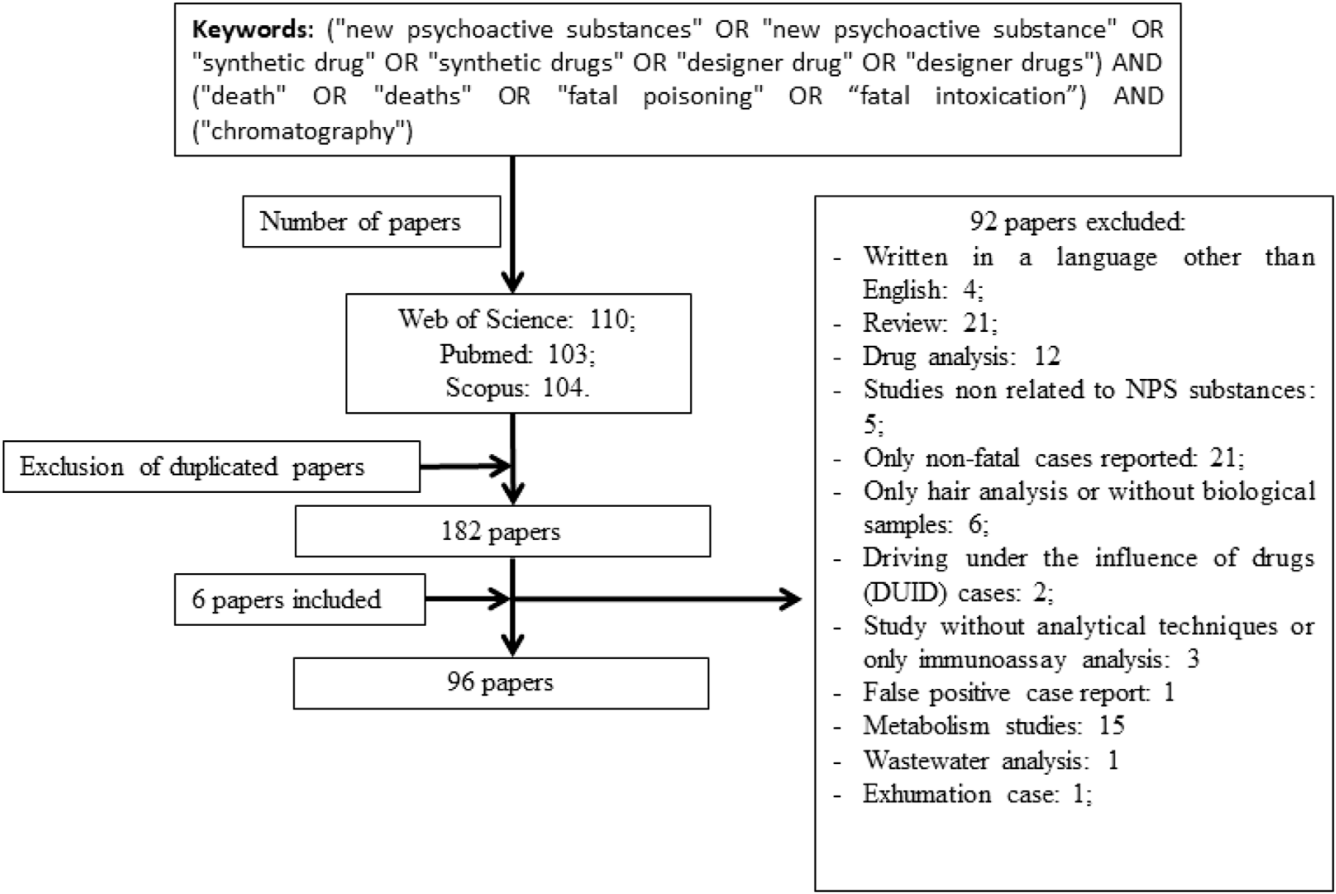
Paper selection strategy.

A literature search was performed on PubMed, Scopus and Web of Science databases for papers related to fatal cases involving NPS using the following keywords (“new psychoactive substances” OR “new psychoactive substance” OR “synthetic drug” OR “synthetic drugs” OR “designer drug” OR “designer drugs”) AND (“death” OR “deaths” OR “fatal poisoning” OR “fatal intoxication”) AND (“chromatography”). Only papers published in English from January 2016 to December 2021 were considered. Additionally, six papers mentioned in some studies that escaped from our search were included. All papers were screened independently by three of the authors and only papers selected by at least two of them were included. The paper selection strategy, including the exclusion criteria, is summarized in Figure 2.

**FIGURE 3:**
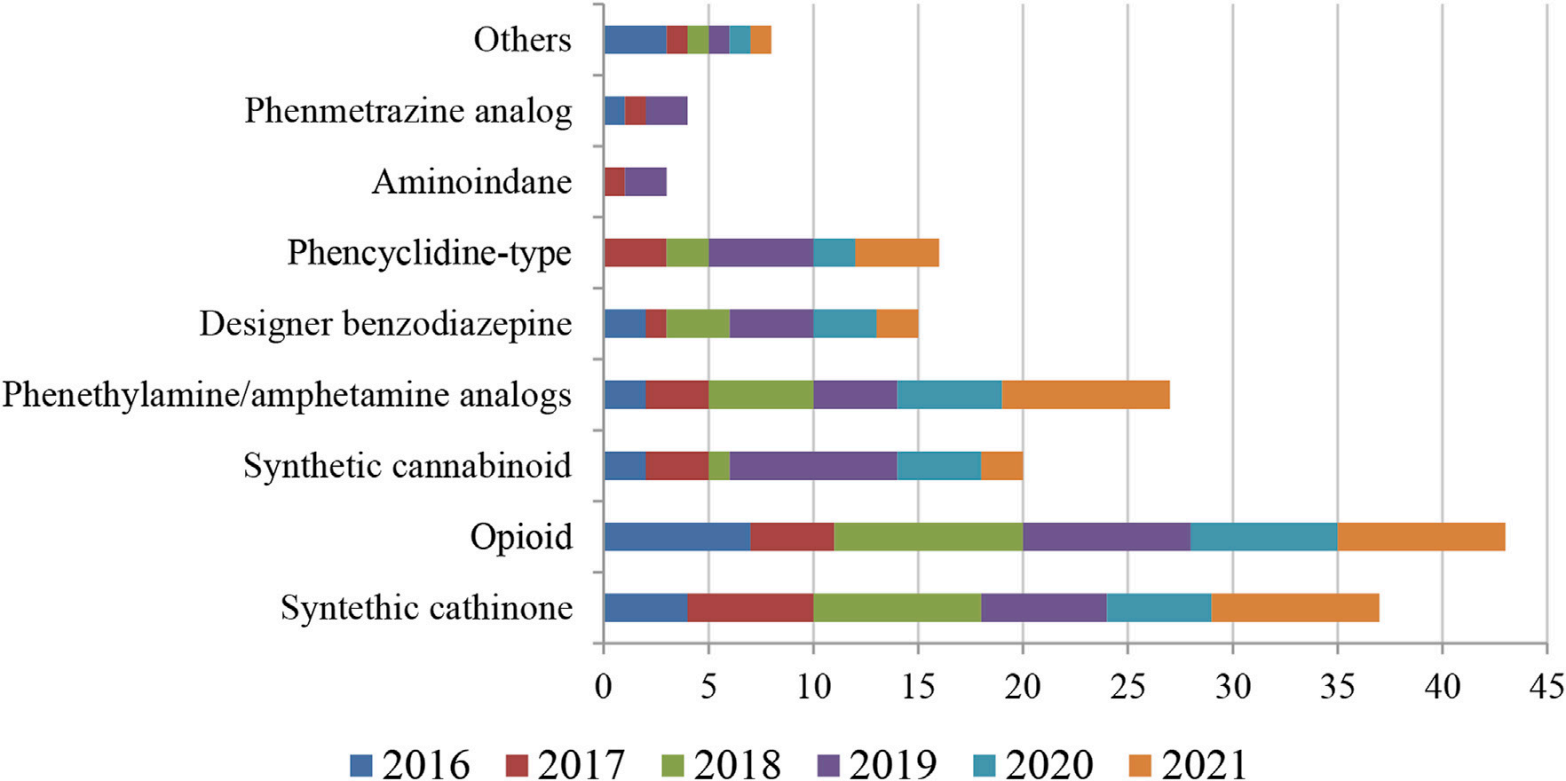
Main NPS classes reported in the publications from 2016 to 2021. Others: piperazine (2016), tryptamine (2017–2021), indole alkaloids found in kratom (2018 and 2019); methaqualone analog (2020). Amphetamine analogs were included in phenethylamine data. Opioid includes fentanyl and analogs.

A total of 96 papers were retrieved for this review, with the highest number found in 2018 and 2019 (20 and 19 papers, respectively). Opioids and synthetic cathinones were the NPS classes most found in the fatal cases, reported in 43 and 37 of the studies, respectively (Figure 3) and this trend was observed in each year. A summary description of all papers is shown in Table 1, and include the analytical technique and extraction/cleanup methods used, the limits of detection and quantification (LOD/LOQ), the main substances found, the concentration range in blood and/or urine and the number of fatal cases. A more detailed description of the studies can be found in Supplementary Table S1.

**TABLE 1:** Summary of results found in the 96 papers included in this review that investigated new psychoactive substances in blood and/or urine from fatal cases. Aditional information of each study can be found in Supplementary Table S1.

References	Extraction method	Analytical techniques	LOD/LOQ (ng/mL or ng/g) ^a^	Substance (class)	Blood/urine, analyte concentration and number of postmortem/death cases^a^
Adamowicz et al. (2016) ^b^	LLE	LC-MS/MS	0.036/1	α-PVP (cath.)	Blood (1.1–6,200); *n* = 12
Beck et al. (2016)	PP	LC-MS/MS (ID, Q); LC-HR/MS (ID)	0.2/-	α-PVP (cath.)	Serum (62.6–304); *n* = 2
Coopman et al. (2016)^b^	LLE	UPLC-MS/MS	2.1/2.1	Ocfentanil (opioid)	Blood (15.3); *n* = 1
Fujita et al. (2016)	QuEChERS	LC-MS/MS	-	Mepirapim (SC); α-EAPP (opioid)	Blood (*n* = 1): Mepirapim (950); α-EAPP (3,100)
Gieron and Adamowicz, (2016)^b^	PP	LC-MS/MS	0.06/0.1	AB-CHMINACA (SC)	Blood (1.5); urine (0.1); *n* = 1
Kristofic et al. (2016)	SPE	LC-QTOF (SCR); LC-MS/MS (Q)	-	25C-NBOMe (PEA)	25C-NBOMe: blood (0.48–2.07), urine (1.73–27.43); 2C-C: blood (0.12), urine (0.11–0.38); *n* = 3
Liveri et al. (2016)^b^	SPE	GC-MS	LOD: Blood/urine (0.002–0.01)/LOQ: Blood (0.4–3); urine: (0.8–6)	MDPV and pentedrone (Cath)	MDPV: blood (46), urine (1,300); pentedrone (mg/L): blood (160), urine (12,000); *n* = 1
Papsun et al. (2016)^b^	LLE	LC-QTOF (SCR); LC-MS/MS (Q)	1/-	MT-45 (Piperazine); Etizolam (D-BZD)	Blood: MT-45 (520); etizolam (35); *n* = 1
Poklis et al. (2016)^b^	SPE	UPLC-MS/MS	-/1	Butyryl Fentanyl (opioid)	Butyryl fentanyl: P. blood (99–3.7), H. blood (220–9.2), urine (64–2); *n* = 2
Rojkiewicz et al. (2016)	LLE	HPLC-MS and GC-MS	7/12	4-FBF (opioid)	Blood (91–112), urine (200–414); *n* = 2
Shanks and Behonick, (2016)^b^	LLE	LC-MS/MS	0.1/0.2	5F-AMB (SC)	Blood (0.3); *n* = 1
Yonemitsu et al. (2016)^b^	QuEChERS	LC-MS/MS and GC-MS (SCR); LC-MS/MS (Q)	-	Acetyl fentanyl (opioid); 4-MeO-PV8 (Cath)	Acetyl fentanyl: F. blood (153), urine (240); 4- MeO-PV8: F. blood (389), urine (245); *n* = 1
Angerer et al. (2017)^b^	LLE	GC-MS, HPLC-MS/MS, HPLC-PDA (SCR); LC-MS/MS (Q)	0.01–0.03/0.1–0.25	5F-PB-22, AB-CHMINACA and 5F-ADB (SC)	F. blood: 5F-PB-22 (0.37), n = 1, AB-CHMINACA (4.1), *n* = 1; 5F-ADB (0.38), *n* = 1
Bottinelli et al. (2017)^b^	SPE	GC-MS, LC-DAD (SCR); GC-MS/MS (Q)	-/50	3-MMC (Cath)	P. blood (249), urine (29,694); *n* = 1
Dwyer et. Al. (2018)	LLE/SPE	GC-MS (SCR); LC-MS/MS (Q)	-	Fentanyl and acetylfentanyl (opioid)	Blood: acetylfentanyl (0.13–2,100); fentanyl (0.24–74.3); urine: only qualitative; *n* = 41
Ellefsen et al. (2017)^b^	LLE	LC-MS/MS and GC-MS	-/0.001	3-FPM (PHEN); U-47700 (opioid)	3-FPM: P. blood (2,400), aortic blood (600); U-47700: P. blood (360); *n* = 1
Guerrieri et al. (2017)^b^	LLE-LTP	LC-MS/MS	-	Acrylfentanyl (opioid)	Blood (0.01–5); *n* = 40
Johansson et al. (2017)^b^	LLE	LC-TOF-MS (SCR); LC-MS/MS (Q)	-/0.01	3-MeO-PCP (PCY)	Blood (50–180); (*n* = 6); blood (380 μg/g) in a mono-intoxication case; *n* = 1
Krotulski et al. (2017)	SPE	LC-QTOF (SCR,MI); LC-MS/MS (Q)	-	THFF and U-49900 (opioid); MeO-PCP (PCY)	Blood and urine, respectively: THFF (339; >5,000); U-49900 (1.5; 2.2); MeO-PCP (1.0; 31.8); *n* = 1
Paul et al. (2017)	NA	LC-MS/MS	0.01–2.0/0.1–2.0	AB-CHMINACA, UR-144, XLR-11 and JWH-022 (SC)	Blood: AB-CHMINACA (8.2), *n* = 1; UR-144 (12.3), XLR-11 (1.3) and JWH-022 (3), *n* = 1
Potocka-banas et al. (2017)	LLE	LC-MS/MS	1/5	α-PVP (Cath)	α-PVP: blood (174), urine (401); *n* = 1
Rojek et al. (2017)^b^	LLE	LC-MS/MS	-/0.05–10	UR-144 (SC); Pentedrone (Cath)	Blood: UR-144 (2.1), *n* = 1; UR-144 (1.4), pentedrone (2,300), (*n* = 1); UR-144 (4), pentedrone (290), *n* = 1
Staeheli et al. (2017)^b^	LLE	LC-MS/MS	-	MDAI (AI); 2-MAPB (Cath)	P. blood: MDAI (38); 2-MAPB (21); *n* = 1
Wiergowski et al. (2017)^b^	PP/LLE	HPLC-QTOF-MS (SCR); UPLC-MS/MS (Q)	0.0053–0.0013/0.0159–4.0	25B-NBOMe (PEA); 4-CMC (Cath)	Blood. 25B-NBOMe (38.4–661), 4-CMC (0.887–2.14); *n* = 2
Allibe et al. (2018)^b^	SPE	LC-MS/MS (ID, Q); LC-HRMS (MI)	0.01/0.05	Ocfentanil (opioid)	Ocfentanil: P. blood (3.7); *n* = 1
Atherton et al. (2018)	LLE	GC-MS	-/10	N-ethylpentylone (Cath)	P. blood (31–953); *n* = 4
Ballesteros et al. (2018)^b^	SPE	LC-MS/MS and GC-MS	20/-	4-MEC and α-PVP (Cath)	α-PVP: blood (9–1,200); urine: detected 4-MEC and α-PVP; *n* = 2
Costa et al. (2018)^b^	LLE	LC-MS/MS	1 and 5/-	N-ethylpentylone (Cath)	Blood (170); *n* = 1
Fagiola et al. (2018)^b^	LLE	GC-MS or LC-MS (SCR); LC-MS/MS	2.5 (LC-MS/MS); 200 (GC–MS or LC-MS, for Cath)/-	Mitragynine and 7-OH-mitragynine; Pentylone, methylone and butylone (Cath)	Blood/urine: Mitragynine, *n* = 2; mitragynine and 7-hydroxymitragynine, *n* = 3; pentylone, methylone and butylone, *n* = 1
Gerace et al. (2018)^b^	LLE	UHPLC-MS/MS	0.6/2	U-47700 (opioid)	Blood (380); urine (10,400); *n* = 1
Koch et al. (2018)^b^	PP/LLE/SPE	LC-MS/MS	-/1	U-47700 (opioid)	Blood: 42 min (370), 9 h (37), 24 h. (6.3), 33 h (2.1), 41 h (2.3); urine (2); *n* = 1
Krpo et al. (2018)	LLE	UHPLC-QTOF-MS (SCR); UHPLC-MS/MS: (ID, Q)	-	5-APB (PEA)	P. blood (860); *n* = 1
Kusano et al. (2018)^c^	PP	LC-MS/MS (SCR, Q) LC-QTOF-MS (SCR)	0.005–0.1/-	Diphenidine (PCY); 5F-ADB (SC)	Blood (*n* = 1): 5F-ADB (0.19 ± 0.04), diphenidine (12 ± 2.6)
Lehmann et al. (2018)^b^	SPE/QuEChERS	LC-MS/MS	0.4–5/-	Methoxetamine (PCY); 4-MEC, MDPV and α-PVP (Cath)	F. blood: 4-MEC (8–118), MDPV (3–396), MXE (2–385) and α-PVP (4); *n* = 2
Maher et al. (2018)	LLE	HPLC-DAD; LC-QTOF-MS (ID); LC-MS/MS (ID, Q)	0.05–0.16/-	Cyclopropylfentanyl and crotonylfentanyl (opioid)	F. blood: (16.6–28.9); *n* = 4
Majchrzak et al. (2018)^c^	LLE	LC-MS/MS	Body fluids: 9.0–27.2; tissues: 15.0–46.0/-	N-PP (Cath)	N-PP: blood (3,100); *n* = 1
Mardal et al. (2018)^b^	LLE/PP	UHPLC-MS/MS (ID, Q); UHPLC-HR-MS/MS (MI)	-/7–68	Methoxyacetylfentanyl (opioid)	F. blood (22–56); *n* = 3
Moody et al. (2018)^b^	SPE	LC–MS/MS (Q); LC-TOF (SCR)	0.0125–0.25/0.05–0.5	4-ANPP, 2-Furanylfentanyl, carfentanil, fluorobutyrylfentanyl, U-47700, acrylfentanyl, butyrylfentanyl, fluorofentanyl, 4-methoxybutyrylfentanyl and valerylfentanyl (opioid)	Blood: 4-ANPP (0.1–410), n = 1,549; 2-furanylfentanyl (0.1–710), *n* = 1,228; carfentanil (0.1–120), *n* = 697; fluorobutyrylfentanyl (0.1–760), *n* = 563; U-47700 (0.2–3,800), *n* = 543; acrylfentanyl (0.1–29), *n* = 266; butyrylfentanyl (0.1–760), *n* = 142; p-fluorofentanyl (0.1–1), *n* = 31; o-fluorofentanyl (2.4), *n* = 1; 4-methoxybutyrylfentanyl (79), *n* = 1; valerylfentanyl (0.44), *n* = 1
Nooble et al. (2018)^b^	PP/SPE	LC-QTOF-MS (SCR); UHPLC-MS/MS: (Q)	1–5/5	Fentanyl (opioid)	Blood: fentanyl (7–39); *n* = 17
Partridge et al. (2018)^b^	LLE	LC-QTOF: (SCR, Q, MI)	0.8–3/-	U-47700 (opioid); Diclazepam and flubromazepam (D-BZD)	P. blood: U-47700 (330), diclazepam (70), flubromazepam (10); *n* = 1
Pieprzyca et al. (2018)^b^	PP	LC-MS/MS	5/10	PV8 (Cath)	PV8: blood (70–260), urine (110–130); *n* = 2
Rohrig et al. (2017)^b^	SPE	GC-MS (SCR); GC-NPD (SCR, Q)	25/-	U-47700 (opioid)	U-47700: H. blood (260), F. blood (400), urine (4,600); *n* = 1
Strehmel et al. (2018)	PP	LC-QTOF-MS (SCR); LC-MS/MS: (Q)	-	U-47700 (opioid)	U-47700 (µg/ml): F. blood (290), H. blood (12,500), urine (240); *n* = 1
Tomczak et al. (2018)^b^	LLE	GC-MS	0.3/1	4-CMC (Cath)	Blood: (56.2–1870); *n* = 6
Adamowicz et al. (2019)^b^	PP	LC-MS/MS	-/0.1	AMB-FUBINACA and EMB-FUBINACA (SC)	AMB-FUBINACA, EMB-FUBINACA, respectively: blood (ND, ND), urine (4.7, 0.2); *n* = 1
Al-Matrouk et al. (2019)	SPE	LC–MS/MS and LC-HRMS (SCR)	-	5F-AB-PINACA, AB-PINACA, AB-CHIMICA, FUB-AMB, 5F-AB-PINACA, 5F-AKB-48, 5Cl-AKB-48, ADB-PINACA and 5F-ADB (SC)	Urine: only qualitative analysis (*n* = 6)
Ameline et al. (2019)^b^	LLE	GC-MS (SCR); UPLC-MS/MS (Q)	-	3-MeO-PCP (PCY)	P. blood (498), CAR (743), urine (16.7); *n* = 1
Chesser et al. (2019)^b^	SPE	LC-MS/MS	0.05–0.1/0.1	4-ANPP, acetylfentanyl, fentanyl, furanylfentanyl, norfentanyl and U-47700 (opioid)	Blood (femoral, cardiac, iliac, subclavian) (0.1–45; 0.1–227; 0.1–98; 0.2–89; 0.1–38; 0.4->500), for 4-ANPP, acetylfentanyl, fentanyl, furanylfentanyl, norfentanyl, U-47700, respectively; *n* = 58
De Jong et al. (2019)	SPE	UPLC-MS/MS (Q); LC-QTOF-MS (SCR)	-	3-MeO-PCP (PCY)	Serum (123), blood (152); *n* = 1
Deville et al. (2019)^b^	LLE	GC-MS and UPLC-TOF-MS (SCR, ID); HPLC-DAD (Q)	-	MDAI (AI); 5-EAPB (Cath)	MDAI, 5-EAPB, 5-MAPB, 5-APB, respectively: blood (2090, 6,450, 89, 546); urine (69,400, 14,800, 1,000, 48,800); *n* = 1
Fagiola et al. (2019)^b^	LLE	LC-MS/MS	2.5/-	Cyclopropylfentanyl (opioid)	CAR (5.6–82); *n* = 5
Fels et al. (2019)^b^	LLE/SPE	LC-QTOF-MS (ID, Q)	5/10	U-47700 (opioid)	U-47700: F. blood (27–2,200), H. blood (39–4,900), urine (100–5,400); *n* = 26
Freni et al. (2019)^b^	SPE	LC-MS/MS	0.03–0.1/-	Furanylfentanyl and 4-ANPP (opioid)	Furanyl fentanyl and 4-ANPP, respectively: CAR (11.8; 93.5), F. blood (2.7; 50.4), urine (71.3; 171.7); *n* = 1
Gaulier et al. (2019)^b^	SPE	LC-QTOF (SCR); LC-MS/MS (Q)	0.05/0.1	Carfentanil (opioid)	Blood (4.20), urine (0.40); *n* = 1
Ivanov et al. (2019)	LLE	GC-MS (ID); HPLC-UV (Q)	5F-ADB 25/-	5F-ADB and FUB-AMB (SC)	5F-ADB: blood (3.7); *n* = 1
Kovács et al. (2019)^b^	LLE	LC-MS/MS	0.01–10/-	N-ethylhexedrone (Cath); ADB-FUBINACA (SC)	Blood: NEH (285), ADB-FUBINACA (0.08); *n* = 1
Kriikku et al. (2019)^b^	SPE	UPLC-TOF-MS (SCR); GC-MS: (Q)	10/20	U-47700 (opioid)	Blood (150–2000), *n* = 10; urine (20–2,200), *n* = 12
Krotulski et al. (2019)	LLE/SPE	LC-QTOF (ID, MI)	-	4F-MDMB-BINACA (SC)	Blood and urine: qualitative analysis; *n* = 20
Lehmann et al. (2019)	SPE/QuEChERS	LC-MS/MS	-	Diclazepam and pyrazolam (D-BZD); 3-FPM (PHEN)	Diclazepam, pyrazolam, 3-FPM, respectively: F. blood (1; 28; 10), H. blood (1; 28; 9), urine (1; 500; 120); *n* = 1
Margasińska-Olejak et al. (2019)^b^	LLE	LC-MS	-	3-MMC (Cath)	Blood (800); *n* = 1
Nash et al. (2019)^b^	LLE	LC-QTOF (SCR, Q)	-	Furanylfentanyl (opioid); MMMP (Cath)	P. blood: furanylfentanyl (1.6), MMMP (6.7); *n* = 1
Theofel et al. (2019)^b^	PP/SPE	LC-MS/MS (Q)	3/5	N-ethyldeschloroketamine (PCY)	N-ethyldeschloroketamine: urine (3,468), H. blood (2,159), F. blood (375); *n* = 1
Yeter and Erol Öztürk, (2019)^b^	SPE	LC – HRMS (ID, Q)	Blood: 0.08; urine: 0.10/blood: 0.10; urine: 0.12	5F-ADB and its methyl ester hydrolysis metabolite (SC)	Blood: 5F-ADB (0.10–1.55), 5F-ADB metabolite (0.15–23.4), *n* = 70; urine: 5F-ADB metabolite (0.28–72.2), *n* = 34
Adamowicz et al. (2020a)	LLE	LC-MS/MS	0.3/5	α-PiHP (Cath)	α-PiHP: blood (69), urine (2072); *n* = 1
Adamowicz et al. (2020b)^b^	LLE	LC-MS/MS	0.01–0.20/-	Benzylfentanyl (opioid)	Blood: Benzylfentanyl (66; 110); fentanyl (31; 32); norfentanyl (22; 41); 4-FiBF (74); despropionyl-4-FF (6.5); *n* = 3
Benedicte et al. (2020)^b^	LLE	GC-MS (SCR); LC-HRMS: (ID, MI)	0.5/1	MPHP and N-ethyl-4′methylpentedrone (Cath)	MPHP and 4-MEAP, respectively: F.blood (47; 1.6), CAR (97; 3.5), urine (2,380; 49,700); *n* = 1
Ditrana et al. (2020)^b^	PP	HPLC-MS/MS	Blood: 0.03–0.35; urine: 0.02–0.25/blood: 0.08–1; urine: 0.06–0.5	Cyclopropylfentanyl, methoxyacetylfentanyl, furanylfentanyl, acetylfentanyl, 4-ANPP and fentanyl (opioid)	Blood (0.2–9); urine (0.2–8,900), for fentanyl derivatives; *n* = 41
Garneau et al. (2020)	SPE	GC-MS (SCR); LC-MS/MS (SCR, Q)	-	4-ANPP, furanylfentanyl, U-47700, p-fluorobutyrylfentanyl, methoxyacetylfentanyl, cyclopropylfentanyl/crotonylfentanyl, acetylfentanyl, despropionyl fluorofentanyl and N-methyl U-47931 E (opioid)	Cardiac and F. blood, respectively: 4-ANPP (33–32; 18), furanylfentanyl (14–2.4; 0.89) and U-47700 (54–45; 26); *n* = 2. Cardiac and F. blood, respectively: 4-ANPP (5.1; 9.7), p-fluorobutyrylfentanyl (31; 27), methoxyacetylfentanyl (70; 14), cyclopropylfentanyl/crotonylfentanyl (0.15; 0.1), only detected: U-47700, acetylfentanyl, despropionyl fluorofentanyl, N-methyl U-47931E; *n* = 1
Hvozdovich et al. (2020)	SPE	LC-MS/MS	-	5F-ADB, FUB-AMB, 5F-AMB, MDMB-FUBINACA, and AB-CHMINACA (SC)	Blood and/or urine: only qualitative analysis; *n* = 54.5F-ADB was the most prevalent substance
Kriikku et al. (2020)^b^	LLE	GC-NCI-MS	1/-	Flualprazolam (D-BZD)	Blood (3.0–68); *n* = 33
Krotulski et al. (2020a)	LLE/SPE	LC-QTOF-MS (SCR, MI)	-	APP-BINACA (SC)	Blood and urine: only qualitative analysis; *n* = 11
Krotulski et al. (2020b)^c^	LLE	LC-MS/MS (Q); LC-QTOF-MS (MI)	<0.02/-	Isotonitazene (opioid)	Blood (0.4–9.5), *n* = 18; urine (0.6–4.0), *n* = 6; *n* = 1
Lehmann et al. (2020)^b^	SPE	LC-MS/MS	0.4–4/5	PMMA, PMA, PMEA, 2-FA, 4-FA, 2-FMA, 3-FPM, 2-DPMP, MDEA, MDMA, MDA and methiopropamine (PEA); 3-MeO-PCP and MXE (PCY); m-CPP (piperazine); MDPBP, MDPV, 4-MEC, methedrone, methylone and α-PVP (Cath); U-47700 (opioid); pyrazolam, diclazepam; delorazepam; lormetazepam (D-BZD)	AMP and analogs (PMMA, PMA, PMEA, 4-FA, 2-FA, 2-FMA, methiopropamine, MDMA, MDA, MDEA, amphetamine, *n* = 13): 4.5–185000 (urine); 2.2–2,500 (blood). M-CPP (*n* = 1): 130 (urine), 5.3 (blood); MXE (*n* = 4): 6.6–22300 (urine), 1–390 (blood); 3-FPM (*n* = 1): 120 (urine), 5.3 (blood); U-47700 (*n* = 1): 1,500 (urine); 2-DPMP (*n* = 1): 52 (urine), 5.2 (blood); 3-MeO-PCE (*n* = 1): 3.6 (urine); BZD: (Pyrazolam, diclazepam, delorazepam, lormetazepam, *n* = 1): 1–100 (blood); Cath: (4-MEC, MDPV, methedrone, methylone, MDPBP, α-PVP, *n* = 4: 6.2–830 (urine). 3.6–340 (blood); *n* = 17
Tiemensma et al. (2020)	NA	GC-MS and LC-MS	-	Cumyl-PEGACLONE (SC)	Blood (0.73–3.0); n = 5
Woods, (2020)^b^	LLE	GC-MS	<10/50	Mebroqualone (Meth)	F. blood (10,228; 115); *n* = 2
Zawadzki et al. (2020a)^b^	LLE	UHPLC-MS/MS	-/0.1	5F-CUMYL-P7AICA (SC)	Blood (2.8), urine (3.1); *n* = 1
Zawadzki et al. (2020b)^b^	LLE	UHPLC-MS/MS	-/1	N-ethylpentylone (Cath)	P. blood (10,600), urine (17,600); *n* = 1
Arbouche et al. (2021)	LLE	LC-MS/MS (Q); LC-HRMS (ID, MI)	-	3-MeO-PCP (PCY)	F. blood (525), urine (384); *n* = 1
Brahan et al. (2021)^b^	LLE	GC-MS/MS	-/1,000	4-MEC (Cath)	4-MEC: P. blood (14,600), CAR (43,400), urine (619,000); *n* = 1
Castellino et al. (2021)	LLE	GC-MS	1.0/-	Cyclopropylfentanyl (opioid)	Blood (14), *n* = 1; Other case: only detected, *n* = 1
Cartiser et al. (2021)^b^	SPE	GC-MS	-	4-MPD (Cath)	4-MPD: P. blood (1,285), CAR (1,128), urine (>10,000); *n* = 1
Chan et al. (2021)^b^	PP	LC-MS/MS	-	Carfentanil (opioid)	P. blood (0.5), (*n* = 1); iliac blood (0.9), *n* = 1
Ferrari Júnior and Caldas (2021)^b^	QuEChERS	UHPLC-MS/MS	4/10	N-ethylpentylone (Cath)	Blood (597); *n* = 1
Gicquel et al. (2021)^b^	SPE	LC-MS/MS (SCR); LC-HRMS (SCR, Q)	5/10	2F-DCK and 3-MeO-PCE (PCY)	2F-DCK, 3-MeO-PCE and 5-MeO-DMT, respectively: P. blood (1780; 90; 52), urine (6,100; 6,300; 2,200); *n* = 1
Hofmann et al. (2021)^b^	PP	HPLC-MS/MS	1.8–2.6/4.6–6	5-APB and 6-APB (PEA)	5-APB and 6-APB, respectively: C. blood (2,400; 660), P. blood (850; 300), urine (8,700; 3,400; *n* = 1
Kronstrand et al. (2021)^b^	PP	LC-MS/MS (Q). LC-QTOF-MS (MI)	-/2	Methoxyacetylfentanyl (opioid)	F. blood: (18–140); *n* = 10
Krotulski et al. (2021a)	LLE and PP/SPE	LC-MS/MS (Q); LC-TOF-MS (SCR); LC-QTOF-MS (MI)	-/1	Eutylone (Cath)	Blood (1,2–11000), *n* = 67; urine (60; 3,400; and >10,000), *n* = 3
Krotulski et al. (2021b)^c^	LLE	LC-MS/MS (Q); LC-QTOF-MS: (MI)	<0.1/-	Brorfine (opioid)	Blood: 0.1–10; *n* = 20
Krotulski et al. (2021c)^b^	LLE	LC-MS/MS (Q); LC-QTOF-MS (SCR, MI)	0.1/0.5	Metonitazene (opioid)	Blood (0.5–33), urine (0.6–46); *n* = 20
Krotulski et al. (2021d)	LLE/SPE	LC-QTOF-MS (ID, MI)	-	MDMB-4en-PINACA, 5F-MDMB-PICA and 4F-MDMB-BINACA (SC)	Blood: qualitative analysis; *n* = 16
Mochizuki et al. (2021)^b^	SPE	LC-LIT-MS: (ID, Q); GC-MS (ID)	0.1–1/-	4-FMC, 4-MeO-α-PVP, 4-F-α-PVP and PV8 (Cath)	4-FMC, 4-MeO-α-PVP, 4-F-α-PVP and PV8, respectively: H. blood (365; 449; 145; 218), F. blood (397; 383; 127; 167); *n* = 1
Mueller et al. (2021)^b^	SPE	UHPLC-MS/MS	0.01/0.05	Isotonitazene (opioid)	Isotonitazene: F. blood (2.28; 0.59; 0.74), CAR (1.7; 1.13; 0.7), urine (1.88; 3.37; 0.19); *n* = 3
Palazzoli et al. (2021)^b^	PP/SPE	LC-MS/MS	0.1–0.5/0.5–1	Mephedrone, DHM and NORMEP (Cath)	Mephedrone, NORMEP and DHM, respectively: F. blood: (1,088; 47.1; 15.5), C. blood (1,632; 50.2; 49.2), urine (4,443; 740.2; 171.9); *n* = 1
Solbeck et al. (2021)^b^	SPE	LC-MS/MS (Q). LC-QTOF-MS, GC-NPD and GC-MS (SCR)	0.05/0.1	Carfentanil (opioid)	Blood (<0.1–9.2); *n* = 160
Theofel et al. (2021)^b^	NA	GC-MS and LC-QTOF-MS/MS (SCR); LC-MS/MS (Q)	-	2-MAPB (Cath)	2-MAPB: urine (167,000), H. blood (16,700), F. blood (7,300); *n* = 1
Zawadzki et al. (2021)^b^	LLE	UHPLC-MS/MS	0.05/0.1	4-FiBF (opioid)	4-FiBF: blood (76.1–257), urine (289–1,000), VH (89.9–150); *n* = 4

^a^ When necessary, concentrations reported in the studies were converted to ng/mL or ng/g to facilitate the comparison among the methods.

^b^ Papers that described validation procedures.

^c^ Papers that described quantitation by standard addition; C-NMR: carbon-13, nuclear magnetic resonance; EI: electron impact ionization; ELISA: enzyme-linked immunoassay; FT-IR: Fourier-transform infrared spectroscopy; GC-IR: gas chromatography–infrared spectroscopy; GC-MS: gas chromatography coupled to mass spectrometry; GC-MS/MS: gas chromatography coupled to tandem mass spectrometry; GC-NCI-MS: gas-chromatography negative-chemical-ionization mass spectrometry; H-NMR: proton nuclear magnetic resonance; HPLC-DAD: high performance liquid chromatography-diode-array detector; HPLC–DAD-FLD: high performance liquid chromatography-diode-array and fluorescence detectors; HPLC-MS/MS: high performance liquid chromatography-tandem mass spectrometry; HPLC-UV: high performance liquid chromatography-ultraviolet detector; HRMS: high-resolution mass spectrometry; ID: identification; LC-DAD: liquid chromatography-diode-array detector; LC-HRMS: liquid chromatography-high-resolution mass spectrometry; LC-MS: liquid chromatography-mass spectrometry; LC-PDA: liquid-chromatography-photodiode array detector; LC-MS/MS: liquid chromatography-tandem mass spectrometry; LC-QTOF-MS: liquid chromatography-quadrupole time-of-flight mass spectrometry; LC–TOF-MS: liquid chromatography-time of flight mass spectrometry: LC-UV: liquid chromatography-ultraviolet detector; MI: metabolite investigation; MRM: multiple reaction monitoring; NMR: nuclear magnetic resonance; NPS: new psychoactive substance; Q: quantification; SCR: screening; SIM: selective ion monitoring; UHPLC-MS/MS: ultra high performance liquid chromatography-tandem mass spectrometry; UHPLC-QTOF-MS: ultra high performance liquid chromatography-quadrupole time-of-flight mass spectrometry; UPLC-MS/MS: ultra performance liquid chromatography-tandem mass spectrometry; UPLC-TOF-MS: ultra performance liquid chromatography-time-of-flight mass spectrometry; UPLC-PDA: ultra performance liquid-chromatography-photodiode array detector; UV-VIS: ultraviolet/visible spectrophotometry. **Extraction methods**: LLE: liquid-liquid extraction; LLE-LTP: liquid-liquid extraction with low-temperature partition; PP: protein precipitation; QuEChERS: quick, easy, cheap, effective, rugged, and safe; SPE: solid phase extraction. **Substances**: 2-FA: 2-Fluoroamphetamine; 2-FMA: 2-Fluoromethamphetamine; 2-Oxo-PCE: N-ethyldeschloroketamine; 3-FPM: 3-fluoro-phenmetrazine; 3-MMC: 3-methylmethcathinone; 4-FA: 4-Fluoroamphetamine; 4-FMA: 4-Fluoromethamphetamine; 4-FBF: 4-fluorobutyrfentanyl; 4-FiBF: 4-fluoroisobutyryl fentanyl; 4-MEAP: N-ethyl-4′methylpentedrone; 4-MEC: 4-methylethcathinone; 4-MPD: 4-methylpentedrone; 5F-MDMB-PINACA: 5F-ADB; α-PiHP: alpha-Pyrrolidinoisohexaphenone; AI: aminoindane; AMP: amphetamine; BZD: benzodiazepine; BZE: benzoylecgonine; Cath: synthetic cathinone; COC: cocaine; D-BZD: designer-benzodiazepine; DHM: dihydro-mephedrone; MDA: methylenedioxyamphetamine; MDMA: methylenedioxymethamphetamine; Meth: Methaqualone analog; MMMP: 2-methyl-4’-(methylthio)-2-morpholinopropiophenone; MAMP: metamphetamine; N-PP: α-propylaminopentiophenone; NA: not available; ND: non-detected; NORMEP; Nor-mephedrone; PCY: phencyclidine analog; PEA: phenethylamine; PHEN: phenmetrazine analog; PMMA: para-methoxymethamphetamine; SC: synthetic cannabinoid; THC-COOH: 11-Nor-9-carboxy-THC; THC: tetrahydrocannabinol; THFF: Tetrahydrofuranylfentanyl. **Biological fluid/tissues:** C. blood: central blood; CAR: cardiac blood; P. blood: peripheral blood; F. blood: femoral blood; H. blood: heart blood.

## Analytical methods applied in toxicological routine analysis

Keeping the NPS screening methods in toxicological laboratories up to date frequently involves challenges, including reference standard availability, method development, lack of information on new illicit drug, and limitation of immunoassays for many NPS (Partridge et al., 2018; Kriikku et al., 2019). Therefore, it is important that toxicology laboratories have different analytical techniques available to minimize possible methodological limitations.

The analytical techniques used in the studies included in this review are liquid chromatography-mass spectrometry (UHPLC-MS/MS, HPLC-MS/MS, LC-MS/MS, UPLC-MS/MS), which were used in most studies (*n* = 75), followed by LC- high resolution mass spectrometry (LC-QTOF-MS, LC-TOF-MS, UPLC-TOF-MS, LC-HRMS (Orbitrap™), UHPLC-HR-MS/MS, UHPLC-QTOF-MS, and UPLC-TOF-MS; *n* = 35), gas chromatography (GC-NPD, GC-MS or GC-MS/MS; *n* = 29), and LC-DAD methods (*n* = 5). Toxicological analysis used in 48 studies (out of 96) applied more than one technique (GC-MS, LC-DAD, LC-HRMS, LC-MS). Most studies included method validation data, which is essential to guarantee the reliability and suitability of the analytical method, and four of them used standard addition, an interesting analytical approach that overcomes the matrix effect and the need for full validation to quantify few samples (Kusano et al., 2018) (Table 1 and Supplementary Table S1).

GC-MS, available in most forensic laboratories, is a robust and easy-to-handle technique. The electron impact ionization provides reproducible information, allowing high confidence in the screening using trustable reference libraries and the selected ion monitoring (SIM) mode analysis can be applied for targeted screening and quantification (Bottinelli et al., 2017; Ballesteros et al., 2018; Dwyer et al., 2018; Brahan et al., 2021). The use of GC-MS, however, has limitations for labile compounds or those that are present at very low concentration in biological samples. Fagiola et al. (2018) related a possible misidentification of 25I-NBOH as 2C-I by GC-MS analysis, which was later found to be due to the analyte breakdown, since 25I-NBOH was detected intact using LC-QTOF-MS (Arantes et al., 2017). The breakdown of 25R-NBOH family compounds in GC-MS analysis was overcome with shortened columns (4 m-length; Ferrari Júnior et al., 2020). GC-MS was not suitable to screen for synthetic cannabinoids (Hvozdovich et al., 2020), fentanyl analogs (Poklis et al., 2016), phenethylamines (Kristofic et al., 2016), and others NPS due to low concentrations detected in intoxications involving these compounds (Ferrari Júnior and Caldas, 2021). Cathinones can exhibit a “poor fragmentation” in the GC-MS, and more specific mass spectra can be obtained using LC-MS or LC-MS/MS (Mochizuki et al., 2021). On the other hand, Arbouche et al. (2021) describe the discrimination of 3-MeO-PCP and 4-MeO-PCP using GC-MS, since they had the same retention time and transitions exhibited in the LC-MS.

LC-MS/MS methods (electrospray ionization) is indeed an important alternative to overcome the GC limitations. The electrospray ionization is considered a soft technique, with little fragmentation of the molecule when compared to electron ionization used in GC-MS (Arantes et al., 2017; Mochizuki et al., 2021). Its multiple reaction monitoring (MRM) mode is ideal for quantitative methods, demonstrating high sensitivity (Poklis et al., 2016; Paul et al., 2017; Staeheli et al., 2017; Pieprzyca et al., 2018; Adamowicz et al., 2020a; Chan et al., 2021) compared to LC-MS and HPLC-DAD (Adamowicz et al., 2020b).

In forensic toxicology, an extraction/cleanup protocol must guarantee the recovery of a wide range of substances with different physicochemical properties, especially when there is no suspicion of the involved substance (Ferrari Júnior and Caldas, 2018). In total, 16 studies included in this review used protein precipitation (PP) as an intermediate or only extraction step, a simple and fast protocol that presents a poor cleanup. Fifty one studies applied liquid-liquid extraction (LLE) using different solvent systems (mostly using alkaline extraction) (Rojkiewicz et al., 2016; Kriikku et al., 2020), 36 used solid phase extraction (SPE) columns, after solvent/buffer addition, enzymatic hydrolysis and/or PP (Rohrig et al., 2017; Garneau et al., 2020) and five studies used QuEChERS (quick, easy, cheap, effective, rugged, and safe) methods (Table 1), which is a combination of LLE and salts and dispersive SPE with primary and secondary amine (PSA) (Fujita et al., 2016; Ferrari Júnior and Caldas, 2021).

LOD/LOQ assessment can demonstrate if a proposed method is suitable for the analysis of NPS that cause effects at low blood concentrations, and both the extraction/cleanup protocol and the analytical instrumentation must be correctly chosen in search of a better sensitivity. Overall, the lowest LOD/LOQ were achieved by LC-MS/MS. In blood, LOQs in the reviewed studies are mostly below 1 ng/ml, such as 0.2 ng/ml for the opioid benzylfentanyl, using LLE (Adamowicz et al., 2020a), 0.05 ng/ml for isotonitazene (Mueller et al., 2021) and 0.1 ng/ml for the synthetic cannabinoid 5F-ADB, the last two using SPE (Yeter and Erol Öztürk, 2019). Chan et al. (2021) did not inform the LOQ of the LC-MS/MS method, but the authors reported the detection of 0.5 ng/ml of the opioid in blood analysis, using protein precipitation.

Using GC-MS, regardless of the extraction protocols used, the determined LOQs in blood were generally higher, such as 10 ng/ml for N-ethylpentylone (Atherton et al., 2018), 400 ng/ml for MDPV and 3,000 ng/ml for pentedrone (Liveri et al., 2016). Solbeck et al. (2021) stated that GC-MS and GC-NPD screening demonstrated insufficient sensitivity for carfentanil, with a LOD of ∼10 ng/ml in blood. Tomczak et al. (2018) reported a LOQ of 1 ng/ml for 4-CMC using LLE followed by GC-MS after derivatization, a step that is time consuming in a routine work (Ferrari Júnior et al., 2020).

Only two studies include method validation data for matrices other than blood and urine, although quantitative information was provided (Supplementary Table S1). The lack of validation is a major limitation of the reported values in gastric content and tissue samples, as they are matrices with higher complexity compared to blood and urine. Using LC-MS/MS, Chesser et al. (2019) reported LOQ of 0.01 ng/g for opioids in brain and vitreous humor and Palazzoli et al. (2021) reported LOQs of 0.5 or 1 ng/ml or ng/g in liver, kidney, bile and hair for mephedrone and its metabolites.

LC enables other high-resolution hyphenated techniques, such as quadrupole time-of-flight mass analyzers (QTOF), Orbitrap™, that features high mass accuracy being a tool for untargeted screening analysis and for structural characterization and identification of unknown compounds (Theofel et al., 2021). The full scan HRMS data may also be performed to NPS metabolite investigations, which can aid in compound identification (Wiergowski et al., 2017; Allibe et al., 2018; Mardal et al., 2018; Moody et al., 2018; Noble et al., 2018; Partridge et al., 2018; Krotulski et al., 2019; Krotulski et al., 2020a). The metabolite identification helps to understand the metabolic pathway, indicate the presence of active/toxic metabolites (e.g., cocaethylene, produced by the concomitant use of cocaine and alcohol) (Atherton et al., 2018). Sometimes, the metabolite may be the only substance detected when the ingested substance has already undergone biotransformation (Yeter and Erol Öztürk, 2019; Ferrari Júnior and Caldas, 2021).

Furthermore, high resolution techniques are important for monitoring the emergence of new substances onto the market. HRMS, however, requires well-skilled experts and it is a more expensive technique. HPLC-DAD is a good screening and quantification technique, however, it needs mass spectral analysis for compound identification (Angerer et al., 2017; Bottinelli et al., 2017; Maher et al., 2018; Deville et al., 2019; Ivanov et al., 2019). Another HPLC-DAD application would be the differentiation of isomers by the UV spectra (Botinelli et al., 2017). The presence of structural isomers is common among different NPSs, which sometimes becomes a challenge for the analyst. Mayer et al. (2018) found identical fragmentation pattern of the two isomers cyclopropylfentanyl and crotonylfentanyl, and they showed similar relative abundances by LC-MS and UHPLC-QTOF-MS. Despite the small retention time differences, UV spectral differentiation was possible using HPLC-DAD, although it would be necessary to run reference standards to mitigate any system variability. Baseline separation of the two isomer was, however, achieved by Fagiola et al. (2019) using LC-MS/MS, which was also used by DiTrana et al. (2020) to analyse cyclopropylfentanyl and its metabolite cyclopropylnorfentanyl.

The difficulty of distinguishing the 3 isomers of methylmethcathinone (2-MMC, 3-MMC and 4-MMC) in a 3-MMC intoxication case report was overcome by HPLC-DAD analysis, with each isomer showing different spectrum profiles (Botinelli et al., 2017). Theofel et al. (2021) used GC-IR and HPLC-QTOF-MS to identify the correct positional isomer of MAPB (2-MAPB, 5-MAPB or 6-MAPB) in a yellow liquid involved in a fatal case, and the results confirmed the presence of 2-MAPB. LC-QTOF-MS, in the low energy range, was also used to distinguish the isomers 3- and 4-MeO-PCP based on the different relative ratios of the fragments 189 and 274 m/z (De Jong et al., 2019). The ion ratio approach was also used by Krpo et al. (2018) to differentiate between the positional isomers 5-APB and 6-APB by UHPLC-QTOF-MS and UHPLC-MS/MS analysis to solve a fatal case.

With the emergence of new substances on the drug market, intoxication cases involving NPS may not be elucidated so quickly, which makes the reanalysis of the data previously acquired by high resolution techniques, such as LC-QTOF-MS, a mean of understanding these unresolved intoxication cases. In Finland, stored TOF-MS data of blood samples were reprocessed and showed two additional U-47700 positive cases (Kriikku et al., 2019). In Australia, initial screening analysis by LC-QTOF-MS of the postmortem peripheral blood detected methylamphetamine, amphetamine and lorazepam, and some months later, retrospective data analysis also detected U-47700, 2,5-dimethoxy-4-chloroamphetamine, diclazepam and flubromazepam, which were also confirmed in the urine samples (Partridge et al., 2018).

## Non-biological material analysis

Seized drug and other materials found near the victim can be an important source of information, guiding the toxicological screening and contributing to NPS discovery. Some papers retrieved in this review did describe the analysis of these materials (e.g., Papsun et al., 2016; Yonemitsu et al., 2016; Botinelli et al., 2017; Al-Matrouk et al., 2019; Deville et al., 2019; Ivanov et al., 2019; Gicquel et al., 2021). The drug characterization is also important to alert toxicology laboratories about possible new drugs on the market. As example, the characterization of synthetic cannabinoids 4F-MDMB-BINACA (Krotulski et al., 2019) and APP-BINACA (Krotulski et al., 2020a) in seized drugs performed by GC-MS, LC-QTOF-MS and NMR, showed the presence of new substances in the American market, which were also confirmed in biological samples.

High purity drugs found on the site is common and can help elucidating a possible accidental overdose. Mueller et al. (2021) reported isotonitazene powder (higher than 95% purity) found on the site, determined by GC–MS and proton NMR. In an intoxication case involving U-47700 abuse, the analysis of the seized powder by LC-DAD and NMR revealed a purity higher than 85% (Strehmel et al., 2018).

Due to the constant change of the NPS market, the reference standard availability is an issue for toxicology laboratories and the use of high purity seized materials can be an alternative during routine work. Rojkiewicz et al. (2016) reported that a powder from a 4-FBF fatal case, was analyzed by UV-VIS, LC-MS (ion trap MS in MS^2^ and MS^3^), FT-IR, GC-MS and NMR and used as a reference material for toxicological screening. Benedicte et al. (2020) used seized drugs (powders) characterized by LC–HRMS and NMR spectroscopy and showed to contain MPHP and 4-MEAP of 85% purity for the determination of these drugs in biological samples from a real case.

## Fatal cases involving new psychoactive substance intake

Table 1 summarizes the concentration range of the main NPS reported in serum/blood and urine samples analyzed in the investigation of the fatal cases reviewed in this paper. In total, 28 opioids, 26 synthetic cathinones, 12 synthetic cannabinoids, 8 phenethylamine/amphetamines, 5 designer benzodiazepines and 5 phencyclidines were detected in blood samples (Table 1). Details of all studies are shown in Supplementary Table S1, including NPS detection in tissues and other matrices and all the substances found in the samples.

Blood is the most used biological fluid to evaluate the function of a drug in modifying human behavior and to investigate intoxication cases, as the blood concentration can be closely correlated with the pharmacological and toxic effects, providing pharmacokinetic data and comparison with the presented clinical signs (Elliott et al., 2018; Ferrari Júnior and Caldas, 2021). Although urine drug concentration should not be used to interpret the effect of a drug on humans, it gives a larger detection window when compared to blood (Ferrari Júnior and Caldas, 2021). Furthermore, in most studies included in this review, the drugs found in blood were detected in urine samples, which also contain the drug metabolites.

In blood and urine, synthetic cannabinoids showed concentrations below 100 ng/ml and, overall, cathinones exhibited the highest concentrations among the reported NPS classes, including eutylone and N-ethylpentylone (higher than 10,000 ng/ml) and 4-methylethcathinone (4-MEC; up to 619,000 ng/ml). Some substances presented a large concentration range in blood from the various studies, as U-47700 (0.2–3,800 ng/ml), 4-chloromethcathinone (4-CMC; 0.887–1870 ng/ml) and N-ethylpentylone (31–10600 ng/ml).

Most studies (67.4%) reported NPS detection along with other substances (Supplementary Table S1), which is very relevant as multiple drugs intake may lead to the interaction among the substances and hinder the identification of the drug or drugs that lead to fatality. Some studies of the main NPS classes are discussed further in this review.

### Opioids

Opioids are a group of drugs comprising a range of substances, including opiates and their synthetic analogues that bind to opioid receptors. Morphine, codeine and thebaine are called opiates, naturally occurring alkaloids found in the opium poppy and their semi-synthetic derivatives include hydrocodone, heroin, oxycodone and buprenorphine. Opioids also include synthetic substances, as methadone, tramadol, fentanyl, and other derivatives (UNODC, 2021c). New synthetic opioids, including fentanyl analogues, have been appearing on the drug market in the last two decades and their extreme potency at very low doses leads to fatal poisonings and have become a problem for both law enforcement authorities and public health professionals, being treated in the United States as an epidemic crisis (UNODC, 2021b). Overall, synthetic opioids were the drug class most found in studies, reported in 43 papers included in this review.

Dwyer et al. (2018) reported 41 deaths involving acetyl fentanyl in Pennsylvania (United States), with the blood concentrations ranging from 0.13 to 2,100 ng/ml. In one case, only the acetyl fentanyl (170 ng/ml) was detected, but in most cases, the deaths were concluded as multiple drug toxicity, including fentanyl (26 blood samples, 0.24–60.9 ng/ml), cocaine, heroin and alcohol.

An Italian fatal intoxication case involving furanyl fentanyl was reported by Freni et al. (2019). A 53-year-old man was found dead with a needle inserted in a vein; a white powder found in the room contained the drug and N-phenetyl-piperidine (4-ANPP), a precursor of the manufacture of fentanyl-type drugs, and also a metabolite. Furanyl fentanyl levels ranged from 2.6 ng/ml in gastric content to 40.1 ng/ml in cerebrospinal fluid (CSF), and 4-ANPP levels ranged from 0.6 (CSF) to 93.5 ng/ml (cardiac blood). The presence of the substances in gastric content indicated not only intravenously but also the oral use of the product.

Maher et al. (2018) determined the synthetic opioid cyclopropylfentanyl in four fatalities that occurred in the United Kingdom, with femoral blood concentrations ranging between 16.6 and 28.9 ng/ml. Cyclopropylfentanyl was deemed to have contributed to death in all four cases, even in the presence of other drugs (not described in the paper). In Italy, cyclopropylfentanyl was detected in 7 postmortem blood (0.8–21 ng/ml) and 11 urine samples (1.3–108 ng/ml). However, the cause of death was not concluded in the study (DiTrana et al., 2020).

Two poisoning cases involving carfentanil in Hong Kong showed blood concentrations of 0.5 and 0.9 ng/ml, and the drugs were indicated as the cause of death (Chan et al., 2021). Carfentanil was detected (>0.05 ng/ml) in 160 Canadian fatal cases, with blood concentrations reaching 9.2 ng/ml (Solbeck et al., 2021); in 156 cases, the deaths were classified as mixed drug toxicity (mainly involving cocaine and fentanyl), and in two cases, only carfentanil was detected in blood at very low concentrations (<0.1–0.84 ng/ml), indicating the high lethality of the drug.

Two studies attributed the cause of death to intoxication by methoxyacetylfentanyl alone or in combination with other drugs in United States of America (10 cases; 18–140 ng/g in blood; Kronstrand et al., 2021) and in Denmark (3 cases, 22–56 ng/g in blood; Mardal et al., 2018). In Italy, methoxyacetylfentanyl were found in postmortem blood (2.5–91 ng/ml) and urine (70–1900 ng/ml), along with its metabolite methoxyacetylnorfentanyl and other synthetic opioids (DiTrana et al., 2020) (Table 1 and Supplementary Table S1). Other studies also described blood concentrations of fentanyl derivatives, including ocfentanil (15.3 ng/ml; Coopman et al., 2016), butyryl fentanyl (99–220 ng/ml; Poklis et al., 2016), 4-fluorobutyrylfentanyl (91–112 ng/ml; Rojkiewicz et al., 2016) and 4-fluoroisobutyryl fentanyl (76.1–257 ng/ml), in addition to synthetic cathinones (N-ethylpentylone, α-PiHP and 4-CMC; Zawadzki et al., 2021).

Reports of fatal cases involving U-47700, a selective agonist of the μ-opioid receptor developed in the 1970s, were retrieved in the search. Rohrig et al. (2017) reported an acute intoxication in United States at levels of 260 and 400 ng/ml in heart and femoral blood, respectively. Vitreous humor, brain, liver and urine showed concentrations ranging from 90 to 4,600 ng/ml. In Canada, the cardiac blood concentration of U-47700 in three fatal cases ranged from 45 to 54 ng/ml (Garneau et al., 2020), along with other opioids. Other cases involving toxic blood levels of U-47700 were also related in Italy (380 ng/ml, blood; Gerace et al., 2018) and Germany (370 ng/ml, blood; Koch et al., 2018), the latter case in association of the benzodiazepine flubromazepam (830 ng/ml).

Recently, a novel opioid class, the benzimidazole derivatives, has been detected in postmortem cases. Mueller et al. (2021) reported 3 fatal cases in Switzerland involving isotonitazene, with concentrations levels ranging from 0.59 to 2.28 ng/ml in blood and from 0.19 to 3.37 ng/ml in urine. Other drugs, including benzodiazepines, were detected within the therapeutic range, and based on circumstantial evidence, autopsy, and toxicological analysis, the death cause was concluded as acute intoxication with isotonitazene. In United States, isotonitazene was found in blood samples from 18 fatal cases, with only the opioid being detected in 9 cases (Krotulski et al., 2020b). The blood concentration ranged from 0.4 to 9.5 ng/ml, similar with those found by Mueller et al. (2021), highlighting that the drug may contribute to the fatal outcome even at low concentrations. After the introduction of isotonitazene, metonitazene and brorphine emerged as potent opioids involved in fatal cases in United States, with concentration in blood (*n* = 20) ranging from 0.5 to 33 ng/ml (metonitazene) and from 0.1 to 10 ng/ml (brorphine) (Krotulski et al., 2021b; 2021c).

### Synthetic cathinones

Khat (*Catha edulis*) is a plant native to Africa and the Arabian Peninsula that contains cathinone, a β-keto amphetamine with mechanism of action similar to amphetamines (Baumann et al., 2018). Although synthetic cathinones are traditionally known as “bath salts”, due to the presentation that was initially sold, these NPS are currently sold in pills, powders, crystals and other formulations.

In France, a case of 3-MMC (3-methylmethcathinone) abuse showed blood concentrations of 249 ng/ml (peripheral) and 609 ng/ml (cardiac) (Botinelli et al., 2017). In another case, a 19-year-old woman died after consuming 3-MMC; levels of 800 ng/ml were found in blood, 153 ng/ml in vitreous humor and 5.5 mg in gastric contents (Margasińska-Olejak et al., 2019).

In Brazil, two fatal cases involving N-ethylpentylone use in rave parties were reported, with postmortem blood concentrations of 170 ng/ml (32 y, man) (Costa et al., 2018) and 597 ng/ml (19 y, woman) (Ferrari Júnior and Caldas 2021). In both cases, the cathinone was the only psychoactive substance detected. This drug has been also associated with other fatal cases worldwide. In Poland, Zawadski et al. (2020b) reported a fatal intoxication of a 30-year-old man, with levels of 10,600 ng/ml in blood and 17,600 ng/ml in urine; in addition to eutylone and four N-ethylpentylone metabolites. Two fatal cases of 34-year-old males involving N-ethylpentylone in United States were reported by Atherton et al. (2018), with levels of 121 and 953 ng/ml in blood; in the first case, other drugs were also found and the cause of death was listed as due to methamphetamine, cocaine, fentanyl, and N-ethylpentylone intoxication.

Three studies reported the detection of synthetic cathinones along with synthetic cannabinoids in fatal cases. In Japan, Fujita et al. (2016) reported serum levels of mepirapim (950 ng/ml) and α-EAPP (α-ethylaminopentiophenone, 3,100 ng/ml). In a Polish fatal case, the synthetic cannabinoid UR-144 and the cathinone pentedrone was found in blood at 4 and 290 ng/ml, respectively, and the death was directly associated with the use of the drugs; two other individuals (UR-144 blood concentration of 2.1 and 1.4 ng/ml) committed suicide, probably due to the psychiatric effects of the drug (Rojek et al., 2017). In a Hungarian fatal case (23-year-old male) involving N-ethyl-hexedrone (NEH, cathinone) and ADB-FUBINACA, showed blood levels of 285 and 0.08 ng/ml, respectively, and five ADB-FUBINACA metabolites (Kovacs et al., 2019). As ADB-FUBINACA concentration was below the toxic level, the authors hypothesized that the cause of death was NEH intoxication, with heart disease being a co-factor.

Other synthetic cathinones were determined in blood/serum from acute intoxications, as shown in Table 1, including eutylone (Krotulski et al., 2021a), N-PP (Majchrzak et al., 2018), 4-MEC (Brahan et al., 2021), α-PVP (1.1–6,200 ng/ml) (Adamowicz et al., 2016; Beck et al., 2016; Potocka-Banas et al., 2017), MPHP (Benedicte et al., 2020), and mephedrone (Palazzoli et al., 2021). A fatal poisoning (20 y, male) after multiple cathinone consumption investigated by Mochizuki et al. (2021) showed concentrations of 4-FMC, 4-MeO-α-PVP, 4-F-α-PVP and PV8 ranging from 145 to 449 ng/ml in heart blood, and from 127 to 397 ng/ml in femoral blood.

### Synthetic cannabinoids

Synthetic cannabinoids are chemically manufactured substances designed to activate endogenous cannabinoids receptors and mimic the psychological effects of THC (Krotulski et al., 2021d), with many groups not structurally related to THC or other natural cannabinoids. Some are still not controlled under international drug control systems and undetected in standard drug screens, characteristics that have contributed to their popularity among drug users.

Herbal mixtures containing the drugs and intended for smoking like marijuana are commonly found in the street drug market, but are also available as bulk powders or soaked or sprayed onto paper to facilitate smuggling into prisons *via* the postal service. In the United States, blood and urine from 54 prisoner fatal overdose cases showed the presence of 5F-ADB, FUB-AMB, 5F-AMB, MDMB-FUBINACA, and AB-CHMINACA (Hvozdovich et al., 2020). Other synthetic cannabinoids were the only drugs detected in 37 cases and were listed as the proximate cause of death.

In Bulgaria, an herbal mixture found in the scene of a fatal case was shown to contain 5F-ADB and FUB-AMB. The 18-years-old victim had been using the herb for several months and overuse it during the last 48 h (Ivanov et al., 2019). Both substances were found in blood and urine, and 5F-ADB blood level was 3.7 ng/ml. The autopsy findings revealed acute respiratory distress syndrome and the authors suggested that the case report could be discussed both as drug-induced and drug-related death resulting from acute intoxication with 5F-ADB and FUB-AMB (Ivanov et al., 2019). 5F-ADB and its methyl ester metabolite was reported by Yeter and Erol Öztürk (2019) in blood (*n* = 70) and urine (*n* = 34) of fatal cases in Turkey with concentrations ranging from 0.10–1.55 ng/ml (5F-ADB, blood), 0.15–23.4 ng/ml (blood, metabolite) and 0.28–72.2 ng/ml (urine, metabolite).

Kusano et al. (2018) also reported the consumption of herbal blend containing 5F-ADB by a Japanese 53-year-old male that resulted in a fatal intoxication. Blood concentrations were 0.19 ng/ml for 5F-ADB and 12 ng/ml for diphenidine, a phencyclidine analog. Investigation of the urinary metabolites revealed pathways involving ester hydrolysis and oxidative defluorination, and further oxidation to the carboxylic acid for 5F-ADB and mono- and di-hydroxylated diphenidine metabolites. The present case demonstrates the importance of urinary metabolite screening for drugs with low blood concentrations.

In Australia, five deaths were related to Cumyl-PEGACLONE use, a synthetic cannabinoid receptor agonist with a gammacarbolinone core (Tiemensma et al., 2020). Levels in postmortem blood ranged from 0.73 to 3.0 ng/ml, but in the case with the highest concentration, the cause of death was also due to acute alcohol intoxication (BAC: 0.24%).

A 29-year-old Polish man was found dead, and the confirmed cause was asphyxia from occlusion of the upper airway by a foreign material (Zawadski et al., 2020a). 5F-CUMYL-P7AICA was detected in blood (2.8 ng/ml) and urine (3.1 ng/ml), but not in the gastric contents. It was suspected that the man smoked the dried plant mixed with the powdered synthetic cannabinoid. No other substance was detected in the screening analysis.

Paul et al. (2017) reported two deaths involving synthetic cannabinoids abuse in United States. Blood analysis found AB-CHMINACA in case 1 (8.2 ng/ml) and UR-144, XLR-11, and JWH-022, in case 2 (12.3, 1.3 and 3 ng/ml, respectively), which, according to the authors, have contributed to the death. A fatal poisoning with AB-CHMINACA and ethanol was reported by Gieron and Adamowicz (2016), with AB-CHMINACA levels ranging from 0.1 (urine) to 2.7 ng/ml (blood from lung). In United States, a herbal incense (Apollo brand) was found with a deceased 34-years-old male and showed to contain 5F-AMB (Shanks and Behonick, 2016). The drug was found at 0.3 ng/ml in blood and as no other substance of toxicological interest was detected, the death was certified as accidental due to synthetic cannabinoid toxicity.

Angerer et al. (2017) reported 3 fatal cases (25–41- year-old males) involving synthetic cannabinoids in Germany. In one case, 5F-PB-22, cannabidiol, traces of AB-CHMINACA and 5F- AKB-48 were detected in the herbal blend ‘Hammer Head’, and 5F-PB-22 was found in the blood at 0.37 ng/ml; the metabolites 5F-PB-22 3- carboxyindole, PB-22 5-hydroxy-pentyl, and PB-22 5-pentanoic acid were detected in the urine. In case 2, the herbal blend ‘Desert Premium Potpourri 2 g’ was found at the scene and shown to contain AB-CHMINACA, which was present in blood at 4.1 ng/ml, and metabolites identified in urine. In case 3, 5F-ADB was found in the seized herbal blend and in blood (0.38 ng/ml); metabolites of 5F-ADB, NE-CHMIMO and MDMB-CHMICA were detected in urine. Considering the death scene, the autopsy and the full toxicological analysis, the authors explained the deaths as consequence of synthetic cannabinoids use, although in the two first cases relevant amounts of ethanol were found in the blood (1.45–2.6 g/kg), which might have contributed to the outcome.

### Postmortem cases involving other substances

Other substances involved in fatal intoxications include phenethylamines, phencyclidine analogues and designer benzodiazepines. Phenethylamines are amphetamine analogues with a phenethylamine core in their structure (Figure 1) and also include ring substituted substances as 2C, NBOMe, NBOH compounds, benzodifurans (e.g., Bromo-Dragonfly) and others (6-APB, PMMA) (Lehmann et al., 2020; UNODC, 2021d). Phencyclidine analogues are N-methyl-d-aspartate (NMDA) receptor antagonist, and include ketamine, 3-MeO-PCP, diphenidine, methoxetamine (MXE), 2F-DCK and 3-MeO-PCE (Lehmann et al., 2018; Arbouche et al., 2021). Designer benzodiazepines include NPS that contain a benzodiazepine core, including structurally closely related compounds and are not controlled under the international drug control system (Lehmann et al., 2019; EMCDDA, 2021b).

Hofmann et al. (2021) reported a fatal case in Germany involving two stereoisomers (5- and 6-(2-aminopropyl)benzofuran), which are substituted benzofuran phenethylamines. Concentrations ranged from 300 to 2,400 ng/ml in blood and from 2,100 ng/ml in bile to 65,000 ng/ml in stomach content. No other substance was detected in the screening and the cause of death was assumed as intoxication with 5-APB/6-APB. In a Norwegian fatal case involving 5-APB, blood analysis showed levels of 860 ng/ml, which was considered the cause of death (Krpo et al., 2018).

A total of 33 fatal cases reported in Sweden and Finland were positive for flualprazolam, a designer benzodiazepine, showing median concentrations of 18.0 (3.0–68 ng/g) and poly-drug use, mainly including opioids, and flualprazolam, which were implicated as the cause of death in 13 cases (Krikku et al., 2020).

Various fatal cases were reported to be due to the use of methoxyphencyclidine (3-MeO-PCP). In Sweden, only the drug was found in femoral blood (380 ng/g) (Johansson et al., 2017), and in the Netherlands, the levels were 123 ng/ml in serum and 152 ng/ml in blood (De Jong et al., 2019). In France, a plastic bag containing 3-MeO-PCP powder was found near a 44 years-old man, and levels of 525 ng/ml were present in femoral blood and of 384 ng/ml in urine, in addition to 6 different metabolites (Arbouche et al., 2021). In another French case, powder and crystals contained 3-MeO-PCP (72.9%) and various catinones were found, and blood concentration of the deceased were 498 ng/ml (peripheral) and 743 ng/ml (cardiac) (Ameline et al., 2019). Gicguel et al. (2021) reported the detection of 3-MeO-PCE (90 ng/ml) in peripheral blood, in addition to **2-**fluorodeschloroketamine (2F-DCK) (1780 ng/ml) and a tryptamine analog, 5-MeO-DMT (52 ng/ml).

A 23-year-old male experienced severe respiratory distress and died after being detained by the police. 25C-NBOMe and 2C-C were detected at levels of 2.07 ng/ml and 27.43 ng/ml (25C-NBOMe) and of 0.12 ng/ml and 0.38 ng/ml (2C-C) in blood and urine, respectively. 25C-NBOMe concentrations in tissues ranged from 15.2 ng/g in liver to 300 ng/ml in gastric contents. Based on case history, autopsy and toxicological findings, the cause of death was 25C-NBOMe toxicity (Kristofic et al., 2016).

Wiergowski et al. (2017) reported an acute intoxication of three young men by 25B-NBOMe and 4-CMC intake. One man died after jumping off the window of the apartment, due to hallucinations; concentrations in the blood were 661 ng/ml (25B-NBOMe) and 0.887 ng/ml (4-CMC). Other man showed strong convulsions, heavy breathing and salivation before dying, and postmortem blood concentrations were 66.5 (25B-NBOMe) and 2.14 ng/ml (4-CMC). The authors concluded that the deaths were due to fatal overdose of 25B-NBOMe; O-demethylathed O, O-bis-demethylathed and glucuronidated metabolites were also found in postmortem blood (Wiergowski et al., 2017).

## Conclusion

A total of 96 papers that reports fatal cases involving NPS published in the literature from 2016 to 2021 were reviewed. LC-MS/MS methods were the most used for quantification analysis, and GC-MS technique was widely used as screening and confirmation method. In addition to screening, high resolution mass spectrometry was the preferred technique used for metabolite identification.

Opioids, synthetic cathinones, phenethylamines/amphetamines and synthetic cannabinoids were the main NPS classes found in the postmortem samples, and polydrug use was reported in most studies, which exposes NPS users to a higher risk of overdose due to potential drug interactions. Furthermore, some drugs, as synthetic cannabinoids and opioids, can be fatal at low doses, making the drug detection and the toxicological evaluation an analytical challenge.

The results of this review indicate that toxicological screening and confirmation methods need to be continuously updated to include new substances that emerge on the drug market. Furthermore, results from non-biological analysis can be a source of information on the possible toxic agent, and provide the laboratory reference material to helping to discover new emerging substances.

## References

[B1] Adamowicz P. Bakhmut Z. Mikolajczyk A. (2020b). Screening procedure for 38 fentanyl analogues and five other new opioids in whole blood by liquid chromatography-tandem mass spectrometry. J. Appl. Toxicol. 40, 1033–1046. 10.1002/jat.3962 32103530

[B2] Adamowicz P. Gieroń J. Gil D. Lechowicz W. Skulska A. Tokarczyk B. (2016). Blood concentrations of α-pyrrolidinovalerophenone (α-PVP) determined in 66 forensic samples. Forensic Toxicol. 34, 227–234. 10.1007/s11419-016-0306-0

[B3] Adamowicz P. Jurczyk A. Gil D. Szustowski S. (2020a). A case of intoxication with a new cathinone derivative α-PiHP – a presentation of concentrations in biological specimens. Leg. Med. 42, 101626. 10.1016/j.legalmed.2019.101626 31751796

[B4] Adamowicz P. Meissner E. Maślanka M. (2019). Fatal intoxication with new synthetic cannabinoids AMB-FUBINACA and EMB-FUBINACA. Clin. Toxicol. 57, 1103–1108. 10.1080/15563650.2019.1580371 30806094

[B5] Al-Matrouk A. Alqallaf M. Alshemmeri A. Bojbarah H. (2019). Identification of synthetic cannabinoids that were seized, consumed, or associated with deaths in Kuwait in 2018 using GC – MS and LC – MS-MS analysis. Forensic Sci. Int. 303, 109960. 10.1016/j.forsciint.2019.109960 31550599

[B6] Allibe N. Richeval C. Phanithavong M. Faure A. Allorge D. Paysant F. (2018). Fatality involving ocfentanil documented by identification of metabolites. Drug Test. Anal. 10, 995–1000. 10.1002/dta.2326 29045066

[B7] Ameline A. Garnier D. Gheddar L. Richeval C. Michel Gaulier J. Sébastien Raul J. (2019). Identification and analytical characterization of seven NPS, by combination of 1 H NMR spectroscopy, GC–MS and UPLC–MS/MS, to resolve a complex toxicological fatal case. Forensic Sci. Int. 298, 140–148. 10.1016/j.forsciint.2019.03.003 30903949

[B8] Angerer V. Jacobi S. Franz F. Auwärter V. Pietsch J. (2017). Three fatalities associated with the synthetic cannabinoids 5F-ADB, 5F-PB-22, and AB-CHMINACA. Forensic Sci. Int. 281, e9–e15. 10.1016/j.forsciint.2017.10.042 29133010

[B9] Arantes L. C. Ferrari Júnior E. de Souza L. F. Cardoso A. C. Alcântara T. L. F. Lião L. M. (2017). 25I-NBOH: A new potent serotonin 5-ht2a receptor agonist identified in blotter paper seizures in Brazil. Forensic Toxicol. 35, 408–414. 10.1007/s11419-017-0357-x 28706567 PMC5486617

[B10] Arbouche N. Kintz P. Zagdoun C. Gheddar L. Raul S. Ameline A. (2021). Determination of 3-MeO-PCP in human blood and urine in a fatal intoxication case, with a specific focus on metabolites identification. Forensic Sci. Res. 1, 208–214. 10.1080/20961790.2021.1928821 PMC8635592 34868712

[B11] Atherton D. Dye D. Robinson C. A. Beck R. (2018). n-Ethyl pentylone-related deaths in Alabama. J. Forensic Sci. 64, 304–308. 10.1111/1556-4029.13823 29768653

[B12] Ballesteros S. Almarza E. Quintela O. Martínez M. A. (2018). The risk of consuming “Bath Salts”. Exemplification through four forensic cases in Spain. Forensic Chem. 11, 87–96. 10.1016/j.forc.2018.10.003

[B13] Baumann M. H. Walters H. M. Niello M. Sitte H. H. (2018). Neuropharmacology of synthetic cathinones. Handb. Exp. Pharmacol. 252, 113–142. 10.1007/164_2018_178 30406443 PMC7257813

[B14] Beck O. Franzén L. Bäckberg M. Signell P. Helander A. (2016). Toxicity evaluation of α-pyrrolidinovalerophenone (α-PVP): Results from intoxication cases within the STRIDA project. Clin. Toxicol. 54, 568–575. 10.1080/15563650.2016.1190979 27412885

[B15] Benedicte L. Camille R. Audrey C. Deborah I. Morgan B. Marie D. (2020). Case report on two-cathinones abuse: MPHP and N-ethyl-4′methylnorpentedrone, with a fatal outcome. Forensic Toxicol. 38, 243–254. 10.1007/s11419-019-00486-x

[B16] Bottinelli C. Cartiser N. Gaillard Y. Boyer B. Bévalot F. (2017). A fatal case of 3-methylmethcathinone (3-MMC) poisoning. Toxicol. Anal. Clinique 29, 123–129. 10.1016/j.toxac.2016.12.010

[B17] Braham M. Y. Franchi A. Cartiser N. Bévalot F. Bottinelli C. Fabrizi H. (2021). Fatal 4-MEC intoxication: Case report and review of literature. Am. J. Forensic Med. Pathol. 42, 57–61. 10.1097/PAF.0000000000000599 32773434

[B18] Cartiser N. Sahy A. Advenier A. S. Franchi A. Revelut K. Bottinelli C. (2021). Fatal intoxication involving 4-methylpentedrone (4-MPD) in a context of chemsex. Forensic Sci. Int. 319, 110659. 10.1016/j.forsciint.2020.110659 33370656

[B19] Castellino C. Cleve D. V. Cabrera R. (2021). Two cyclopropyl fentanyl case studies in los angeles. J. Anal. Toxicol. 45, 105–109. 10.1093/jat/bkaa037 32347303

[B20] Chan W. Wong G. F. Lee W. (2021). Carfentanil related death first encountered in Hong Kong: Two case reports. Forensic Sci. Int. Rep. 3, 100181. 10.1016/j.fsir.2021.100181

[B21] Chesser R. Pardi J. Concheiro M. Cooper G. (2019). Distribution of synthetic opioids in postmortem blood, vitreous humor and brain. Forensic Sci. Int. 305, 109999. 10.1016/j.forsciint.2019.109999 31671355

[B22] Coopman V. Cordonnier J. De Leeuw M. Cirimele V. (2016). Ocfentanil overdose fatality in the recreational drug scene. Forensic Sci. Int. 266, 469–473. 10.1016/j.forsciint.2016.07.005 27471990

[B23] Costa J. L. Cunha K. F. Lanaro R. Cunha R. L. Walther D. Baumann M. H. (2018). Analytical quantification, intoxication case series, and pharmacological mechanism of action for N-ethylnorpentylone (N-ethylpentylone or ephylone). Drug Test. Anal. 11, 461–471. 10.1002/dta.2502 30207090 PMC7316160

[B24] De Jong L. A. A. Olyslager E. J. H. Duijst W. L. J. M. (2019). The risk of emerging new psychoactive substances: The first fatal 3-MeO-PCP intoxication in The Netherlands. J. Forensic Leg. Med. 65, 101–104. 10.1016/j.jflm.2019.05.011 31129558

[B25] Deville M. Dubois N. Cieckiewicz E. De Tullio P. Lemaire E. Charlier C. (2019). Death following consumption of MDAI and 5-EAPB. Forensic Sci. Int. 299, 89–94. 10.1016/j.forsciint.2019.03.023 30981086

[B26] Di Trana A. Mannocchi G. Pirani F. La Maida N. Gottardi M. Pichini S. (2020). A comprehensive HPLC – MS-MS screening method for 77 new psychoactive substances, 24 classic drugs and 18 related metabolites in blood, urine and oral fluid. J. Anal. Toxicol. 44, 769–783. 10.1093/jat/bkaa103 32816015

[B27] Dwyer J. B. Janssen J. Luckasevic T. M. Williams K. E. (2018). Report of increasing overdose deaths that include acetyl fentanyl in multiple counties of the southwestern region of the commonwealth of Pennsylvania in 2015–2016. J. Forensic Sci. 63, 195–200. 10.1111/1556-4029.13517 28605020

[B28] Ellefsen K. N. Taylor E. A. Simmons P. Willoughby V. Hall B. J. (2017). Multiple drug-toxicity involving novel psychoactive substances, 3-Fluorophenmetrazine and U-47700. J. Anal. Toxicol. 41, 765–770. 10.1093/jat/bkx060 28985320

[B29] Elliott S. P. Stephen D. W. S. Paterson S. (2018). The United Kingdom and Ireland association of forensic toxicologists forensic toxicology laboratory guidelines (2018). Sci. Justice 58, 335–345. 10.1016/j.scijus.2018.05.004 30193659

[B30] EMCDDA (2021a). European drug report - trends and developments. Available online at: https://www.emcdda.europa.eu/publications/edr/trends-developments/2021_en (Accessed June 10, 2022).

[B31] EMCDDA (2021b). New benzodiazepines in Europe – a review. Available online at: https://www.emcdda.europa.eu/publications/rapid-communications/new-benzodiazepines-europe-review_en (Accessed June 10, 2022).

[B32] Fagiola M. Hahn T. Avella J. (2019). Five postmortem case reports with qualitative analysis of cyclopropylfentanyl by LC-MS-MS. J. Anal. Toxicol. 43, e1–e6. 10.1093/jat/bky094 30476101

[B33] Fagiola M. Hahn T. Avella J. (2018). Screening of novel psychoactive substances in postmortem matrices by liquid chromatography–tandem mass spectrometry (LC–MS-MS). J. Anal. Toxicol. 42, 562–569. 10.1093/jat/bky050 30371846

[B34] Fels H. Lottner-Nau S. Sax T. Roider G. Graw M. Auwärter V. (2019). Postmortem concentrations of the synthetic opioid U-47700 in 26 fatalities associated with the drug. Forensic Sci. Int. 301, e20–e28. 10.1016/j.forsciint.2019.04.010 31097357

[B35] Ferrari Júnior E. Arantes L. C. Salum L. B. Caldas E. D. (2020). Analysis of non-derivatized 2-(4-R-2, 5-dimethoxyphenyl)-N-[(2-hydroxyphenyl)methyl] ethanamine using short column gas chromatography – mass spectrometry. J. Chromatogr. A 1634, 461657. 10.1016/j.chroma.2020.461657 33161196

[B36] Ferrari Júnior E. Caldas E. D. (2021). Determination of new psychoactive substances and other drugs in postmortem blood and urine by UHPLC–MS/MS: Method validation and analysis of forensic samples. Forensic Toxicol. 40, 88–101. 10.1007/s11419-021-00600-y 36454493

[B37] Ferrari Júnior E. Caldas E. D. (2018). Simultaneous determination of drugs and pesticides in postmortem blood using dispersive solid-phase extraction and large volume injection-programmed temperature vaporization-gas chromatography-mass spectrometry. Forensic Sci. Int. 290, 318–326. 10.1016/j.forsciint.2018.07.031 30121553

[B38] Freni F. Pezzella S. Vignali C. Moretti M. Cisini S. Rossetti C. (2019). A case report on potential postmortem redistribution of furanyl fentanyl and 4-ANPP. Forensic Sci. Int. 304, 109915. 10.1016/j.forsciint.2019.109915 31416646

[B39] Fujita Y. Koeda A. Fujino Y. Onodera M. Kikuchi S. Niitsu H. (2016). Clinical and toxicological findings of acute intoxication with synthetic cannabinoids and cathinones. Acute Med. Surg. 3, 230–236. 10.1002/ams2.182 29123790 PMC5667234

[B40] Garneau B. Desharnais B. Beauchamp-doré A. Lavallée C. Mireault P. Lajeunesse A. (2020). Challenges related to three cases of fatal intoxication to multiple novel synthetic opioids. J. Anal. Toxicol. 44, 86–91. 10.1093/jat/bkz018 30927001

[B41] Gaulier J-M. Richeval C. Phanithavong M. Brault S. Allorge D. Dumestre-Toulet V. (2019). A case report of carfentanil-related fatality in France. Toxicol. Anal. Clinique 31, 323–331. 10.1016/j.toxac.2019.01.002

[B42] Gerace E. Salomone A. Luciano C. Di Corcia D. Vincenti M. (2018). First case in Italy of fatal intoxication involving the new opioid U-47700. Front. Pharmacol. 9, 747. 10.3389/fphar.2018.00747 30042684 PMC6048284

[B43] Gicquel T. Richeval C. Mesli V. Gish A. Hakim F. Pelletier R. (2021). Fatal intoxication related to two new arylcyclohexylamine derivatives (2F-DCK and 3-MeO-PCE). Forensic Sci. Int. 324, 110852. 10.1016/j.forsciint.2021.110852 34049075

[B44] Gieroń J. Adamowicz P. (2016). Fatal poisoning with the synthetic cannabinoid ab-chminaca and ethyl alcohol – a case study and literature review. Problems Forensic Sci. 106, 482–495.

[B45] Guerrieri A. D. Rapp E. Roman M. Thelander G. Kronstrand R. (2017). Acrylfentanyl: Another new psychoactive drug with fatal consequences. Forensic Sci. Int. 277, e21–e29. 10.1016/j.forsciint.2017.05.010 28587915

[B46] Hofmann V. Sundermann T. R. Landmann A. Rechtsteiner S. Schmitt G. Bartel M. (2021). Simultaneous determination of 5- and 6-APB in blood, other body fluids, hair and various tissues by HPLC-MS/MS. J. Anal. Toxicol. 46, 264–269. 10.1093/jat/bkab018 33576419

[B47] Hvozdovich J. A. Chronister C. W. Logan B. K. Goldberger B. A. (2020). Case report: Synthetic cannabinoid deaths in state of Florida prisoners. J. Anal. Toxicol. 44, 298–300. 10.1093/jat/bkz092 31776572

[B48] Ivanov I. D. Stoykova S. Ivanova E. Vlahova A. Burdzhiev N. Pantcheva I. (2019). A case of 5F-ADB/FUB-AMB abuse: Drug-induced or drug-related death? Forensic Sci. Int. 297, 372–377. 10.1016/j.forsciint.2019.02.005 30850157

[B49] Johansson A. Lindstedt D. Roman M. Thelander G. Nielsen E. I. Lennborn U. (2017). A non-fatal intoxication and seven deaths involving the dissociative drug 3-MeO-PCP. Forensic Sci. Int. 275, 76–82. 10.1016/j.forsciint.2017.02.034 28324770

[B50] Koch K. Auwärter V. Hermanns-Clausen M. Wilde M. Neukamm M. A. (2018). Mixed intoxication by the synthetic opioid U-47700 and the benzodiazepine flubromazepam with lethal outcome: Pharmacokinetic data. Drug Test. Anal. 10, 1336–1341. 10.1002/dta.2391 29637722

[B51] Kovács K. Kereszty É. Berkecz R. Tiszlavicz L. Sija É. Körmöczi T. (2019). Fatal intoxication of a regular drug user following N-ethyl-hexedrone and ADB-FUBINACA consumption. J. Forensic Leg. Med. 65, 92–100. 10.1016/j.jflm.2019.04.012 31128567

[B52] Kriikku P. Pelander A. Rasanen I. Ojanperä I. (2019). Toxic lifespan of the synthetic opioid U-47, 700 in Finland verified by re-analysis of UPLC-TOF-MS data. Forensic Sci. Int. 300, 85–88. 10.1016/j.forsciint.2019.04.030 31082566

[B53] Kriikku P. Rasanen I. Ojanperä I. Thelander G. Kronstrand R. Vikingsson S. (2020). Femoral blood concentrations of flualprazolam in 33 postmortem cases. Forensic Sci. Int. 307, 110101–110113. 10.1016/j.forsciint.2019.110101 31865266

[B54] Kristofic J. J. Chmiel J. D. Jackson G. F. Vorce S. P. Holler J. M. Robinson S. L. (2016). Detection of 25C-NBOMe in three related cases. J. Anal. Toxicol. 40, 466–472. 10.1093/jat/bkw035 27206645

[B55] Kronstrand R. Åstrand A. Watanabe S. Gréen H. Vikingsson S. (2021). Circumstances, postmortem findings, blood concentrations and metabolism in a series of methoxyacetylfentanyl-related deaths. J. Anal. Toxicol. 4, 760–771. 10.1093/jat/bkab053 PMC8446433 34009362

[B56] Krotulski A. J. Cannaert A. Stove C. Logan B. K. (2021d). The next generation of synthetic cannabinoids: Detection, activity, and potential toxicity of pent-4en and but-3en analogues including MDMB-4en-PINACA. Drug Test. Anal. 13, 427–438. 10.1002/dta.2935 32997377

[B57] Krotulski A. J. Mohr A. L. A. Diamond F. X. Logan B. K. (2020a). Detection and characterization of the new synthetic cannabinoid APP-BINACA in forensic casework. Drug Test. Anal. 12, 136–144. 10.1002/dta.2698 31788963

[B58] Krotulski A. J. Mohr A. L. A. Kacinko S. L. Fogarty M. F. Shuda S. A. Diamond F. X. (2019). 4F-MDMB-BINACA: A new synthetic cannabinoid widely implicated in forensic casework. J. Forensic Sci. 64, 1451–1461. 10.1111/1556-4029.14101 31260580

[B59] Krotulski A. J. Papsun D. M. Carolina M. S. Kacinko S. L. Logan B. K. (2021b). Brorphine — investigation and quantitation of a new potent synthetic opioid in forensic toxicology casework using liquid chromatography-mass spectrometry. J. Forensic Sci. 6, 664–676. 10.1111/1556-4029.14623 33201526

[B60] Krotulski A. J. Papsun D. M. Chronister C. W. Homan J. Michele M. Hoyer J. (2021a). Eutylone intoxications-an emerging synthetic stimulant in forensic investigations. J. Anal. Toxicol. 45, 8–20. 10.1093/jat/bkaa113 33325503

[B61] Krotulski A. J. Papsun D. M. Friscia M. Swartz J. L. Holsey B. D. Logan B. K. (2017). Fatality following ingestion of tetrahydrofuranylfentanyl, U-49900 and methoxy-phencyclidine. J. Anal. Toxicol. 42, e27–e32. 10.1093/jat/bkx092 29186585

[B62] Krotulski A. J. Papsun D. M. Kacinko S. L. Logan B. K. (2020b). Isotonitazene quantitation and metabolite discovery in authentic forensic casework. J. Anal. Toxicol. 44, 521–530. 10.1093/jat/bkaa016 32091095

[B63] Krotulski A. J. Papsun D. M. Walton S. E. Logan B. K. (2021c). Metonitazene in the United States — forensic toxicology assessment of a potent new synthetic opioid using liquid chromatography mass spectrometry. Drug Test. Anal. 13, 1697–1711. 10.1002/dta.3115 34137194

[B64] Krpo M. Luytkis H. C. Haneborg A. M. Høiseth G. (2018). A fatal blood concentration of 5-APB. Forensic Sci. Int. 291, e1–e3. 10.1016/j.forsciint.2018.08.044 30228015

[B65] Kusano M. Zaitsu K. Taki K. Hisatsune K. Nakajima J. Moriyasu T. (2018). Fatal intoxication by 5F–ADB and diphenidine: Detection, quantification, and investigation of their main metabolic pathways in humans by LC/MS/MS and LC/Q-TOFMS. Drug Test. Anal. 10, 284–293. 10.1002/dta.2215 28544560

[B66] Lehmann S. Kieliba T. Thevis M. Rothschild M. A. Mercer-Chalmers-Bender K. (2020). Fatalities associated with NPS stimulants in the Greater Cologne area. Int. J. Leg. Med. 134, 229–241. 10.1007/s00414-019-02193-z 31735981

[B67] Lehmann S. Schulze B. Thomas A. Kamphausen T. Thevis M. Rothschild M. A. (2018). Organ distribution of 4-MEC, MDPV, methoxetamine and α-PVP: Comparison of QuEChERS and SPE. Forensic Toxicol. 36, 320–333. 10.1007/s11419-018-0408-y

[B68] Lehmann S. Sczyslo A. Froch-Cortis J. Rothschild M. A. Thevis M. Andresen-Streichert H. (2019). Organ distribution of diclazepam, pyrazolam and 3-fluorophenmetrazine. Forensic Sci. Int. 303, 109959. 10.1016/j.forsciint.2019.109959 31546164

[B69] Liveri K. Constantinou M. A. Afxentiou M. Kanari P. (2016). A fatal intoxication related to MDPV and pentedrone combined with antipsychotic and antidepressant substances in Cyprus. Forensic Sci. Int. 265, 160–165. 10.1016/j.forsciint.2016.02.017 26930452

[B70] Maher S. Elliott S. P. George S. (2018). The analytical challenges of cyclopropylfentanyl and crotonylfentanyl: An approach for toxicological analysis. Drug Test. Anal. 10, 1483–1487. 10.1002/dta.2417 29803198

[B71] Majchrzak M. Celiński R. Kowalska T. Sajewicz M. (2018). Fatal case of poisoning with a new cathinone derivative: α-Propylaminopentiophenone (N-PP). Forensic Toxicol. 36, 525–533. 10.1007/s11419-018-0417-x 29963213 PMC6002430

[B72] Mardal M. Johansen S. S. Davidsen A. B. Telving R. Jornil J. R. Dalsgaard P. W. (2018). Postmortem analysis of three methoxyacetylfentanyl-related deaths in Denmark and *in vitro* metabolite profiling in pooled human hepatocytes. Forensic Sci. Int. 290, 310–317. 10.1016/j.forsciint.2018.07.020 30107329

[B73] Margasińska-Olejak J. Celiński R. Fischer A. Stojko J. (2019). A fatal case of poisoning of a 19-year-old after taking 3-MMC. Forensic Sci. Int. 300, e34–e37. 10.1016/j.forsciint.2019.02.040 31056341

[B74] Mochizuki A. Adachi N. Shojo H. (2021). Detection of 4-FMC, 4-MeO-α-PVP, 4-F-α-PVP, and PV8 in blood in a forensic case using liquid chromatography–electrospray ionization linear ion trap mass spectrometry. Forensic Sci. Int. 325, 110888. 10.1016/j.forsciint.2021.110888 34186472

[B75] Moody M. T. Diaz S. Shah P. Papsun D. Logan B. K. (2018). Analysis of fentanyl analogs and novel synthetic opioids in blood, serum/plasma, and urine in forensic casework. Drug Test. Anal. 10, 1358–1367. 10.1002/dta.2393 29633785

[B76] Mueller F. Bogdal C. Pfeiffer B. Andrello L. Ceschi A. Thomas A. (2021). Isotonitazene: Fatal intoxication in three cases involving this unreported novel psychoactive substance in Switzerland. Forensic Sci. Int. 320, 110686. 10.1016/j.forsciint.2021.110686 33497988

[B77] Nash C. Butzbach D. Stockham P. Scott T. Abroe G. Painter B. (2019). A fatality involving furanylfentanyl and MMMP, with presumptive identification of three MMMP metabolites in urine. J. Anal. Toxicol. 43, 291–298. 10.1093/jat/bky099 30566582

[B78] Noble C. Weihe Dalsgaard P. Stybe Johansen S. Linnet K. (2018). Application of a screening method for fentanyl and its analogues using UHPLC-QTOF-MS with data-independent acquisition (DIA) in MSE mode and retrospective analysis of authentic forensic blood samples. Drug Test. Anal. 10, 651–662. 10.1002/dta.2263 28834382

[B79] Palazzoli F. Santunione A. L. Verri P. Vandelli D. Silingardi E. (2021). Post-mortem distribution of mephedrone and its metabolites in body fluids and organ tissues of an intoxication case. J. Pharm. Biomed. Anal. 201, 114093. 10.1016/j.jpba.2021.114093 33957364

[B80] Papsun D. Krywanczyk A. Vose J. C. Bundock E. A. Logan B. K. (2016). Analysis of MT-45, a novel synthetic opioid, in human whole blood by LC-MS-MS and its identification in a drug-related death. J. Anal. Toxicol. 40, 313–317. 10.1093/jat/bkw012 27091064

[B81] Partridge E. Trobbiani S. Stockham P. Charlwood C. Kostakis C. (2018). A case study involving U-47700, diclazepam and flubromazepam - application of retrospective analysis of HRMS data. J. Anal. Toxicol. 42, 655–660. 10.1093/jat/bky039 29945197

[B82] Paul A. B. M. Simms L. Amini S. Paul A. E. (2017). Teens and spice: A review of adolescent fatalities associated with synthetic cannabinoid use. J. Forensic Sci. 63, 1321–1324. 10.1111/1556-4029.13704 29194599

[B83] Pieprzyca E. Skowronek R. Korczyńska M. Kulikowska J. Chowaniec M. (2018). A two fatal cases of poisoning involving new cathinone derivative PV8. Leg. Med. 33, 42–47. 10.1016/j.legalmed.2018.05.002 29778973

[B84] Poklis J. Poklis A. Wolf C. Hathaway C. Arbefeville E. Chrostowski L. (2016). Two fatal intoxications involving butyryl fentanyl. J. Anal. Toxicol. 40, 703–708. 10.1093/jat/bkw048 27339481 PMC5048708

[B85] Potocka-Banaś B. Janus T. Majdanik S. Banaś T. Dembińska T. Borowiak K. (2017). Fatal intoxication with α-PVP, a synthetic cathinone derivative. J. Forensic Sci. 62, 553–556. 10.1111/1556-4029.13326 28028802

[B86] Rohrig T. P. Miller S. A. Baird T. R. (2017). U-47700: A not so new opioid. J. Anal. Toxicol. 42, e12–e14. 10.1093/jat/bkx081 29040568

[B87] Rojek S. Korczyńska-Albert M. Kulikowska J. Kłys M. (2017). New challenges in toxicology of new psychoactive substances exemplified by fatal cases after UR-144 and UR-144 with pentedrone administration determined by LC-ESI-MS-MS in blood samples. Arch. Med. Sadowej Kryminol. 67, 104–120. 10.5114/amsik.2017.71452 29363897

[B88] Rojkiewicz M. Majchrzak M. Celiński R. Kuś P. Sajewicz M. (2016). Identification and physicochemical characterization of 4-fluorobutyrfentanyl (1-((4-fluorophenyl)(1-phenethylpiperidin-4-yl)amino)butan-1-one, 4-FBF) in seized materials and post-mortem biological samples. Drug Test. Anal. 9, 405–414. 10.1002/dta.2135 27863134

[B89] Shanks K. G. Behonick G. S. (2016). Death after use of the synthetic cannabinoid 5F-AMB. Forensic Sci. Int. 262, e21–e24. 10.1016/j.forsciint.2016.03.004 27017174

[B90] Solbeck P. Woodall K. L. Martin T. L. (2021). Strategic decision-making by a forensic toxicology laboratory in response to an emerging NPS: Detection, quantitation and interpretation of carfentanil in death investigations in ontario, Canada, july 2017 to june 2018. J. Anal. Toxicol. 45, 813–819. 10.1093/jat/bkab079 34166495

[B91] Staeheli S. N. Boxler M. I. Oestreich A. Marti M. Gascho D. Bolliger S. A. (2017). Postmortem distribution and redistribution of MDAI and 2-MAPB in blood and alternative matrices. Forensic Sci. Int. 279, 83–87. 10.1016/j.forsciint.2017.08.007 28850871

[B92] Strehmel N. Dümpelmann D. Vejmelka E. Strehmel V. Roscher S. Scholtis S. (2018). Another fatal case related to the recreational abuse of U-47700. Forensic Sci. Med. Pathol. 14, 531–535. 10.1007/s12024-018-0018-3 30229428

[B93] Theofel N. Budach D. Vejmelka E. Scholtis S. Tsokos M. (2021). Toxicological investigations in a death involving 2-MAPB. Forensic Sci. Med. Pathol. 17, 317–321. 10.1007/s12024-021-00366-0 33877515

[B94] Theofel N. Möller P. Vejmelka E. Kastner K. Roscher S. Scholtis S. (2019). A fatal case involving N -ethyldeschloroketamine (2-oxo-PCE) and venlafaxine. J. Anal. Toxicol. 43, e2–e6. 10.1093/jat/bky063 30365028

[B95] Tiemensma M. Rutherford J. D. Scott T. Karch S. (2020). Emergence of cumyl-PEGACLONE-related fatalities in the northern territory of Australia. Forensic Sci. Med. Pathol. 17, 3–9. 10.1007/s12024-020-00334-0 33185835

[B96] Tomczak E. Woźniak M. K. Kata M. Wiergowski M. Szpiech B. Biziuk M. (2018). Blood concentrations of a new psychoactive substance 4-chloromethcathinone (4-CMC) determined in 15 forensic cases. Forensic Toxicol. 36, 476–485. 10.1007/s11419-018-0427-8 29963211 PMC6002423

[B97] UNODC (2020). Current NPS threats, Vol. III. https://www.unodc.org/documents/scientific/Current_NPS_Threats_Vol.3.pdf.

[B98] UNODC (2021a). Early warning advisory on new psychoactive substances. Available online at: https://www.unodc.org/LSS/Page/NPS (Accessed June 10, 2022).

[B99] UNODC (2021d). NPS Substance groups. Available online at: https://www.unodc.org/LSS/SubstanceGroup/GroupsDashboard?testType=NPS (Accessed June 12, 2022).

[B100] UNODC (2021b). World drug report. Drug market trends: Opioids, cannabis. Available online at: https://www.unodc.org/unodc/en/data-and-analysis/wdr2021.html (Accessed September 11, 2022).

[B101] UNODC (2021c). World drug report. Executive summary – policy implications. Available online at: https://www.unodc.org/unodc/en/data-and-analysis/wdr2021.html (Accessed June 10, 2022).

[B102] Wiergowski M. Aszyk J. Kaliszan M. Wilczewska K. Anand J. S. Kot-Wasik A. (2017). Identification of novel psychoactive substances 25B-NBOMe and 4-CMC in biological material using HPLC-Q-TOF-MS and their quantification in blood using UPLC–MS/MS in case of severe intoxications. J. Chromatogr. B Anal. Technol. Biomed. Life Sci. 1042, 1–10. 10.1016/j.jchromb.2016.12.018 27992785

[B103] Woods K. M. (2020). Two fatalities involving mebroqualone. J. Anal. Toxicol. 45, 308–311. 10.1093/jat/bkaa077 32789477

[B104] Yeter O. Erol Öztürk Y. (2019). Detection and quantification of 5F-ADB and its methyl ester hydrolysis metabolite in fatal intoxication cases by liquid chromatography–high resolution mass spectrometry. Forensic Sci. Int. 302, 109866. 10.1016/j.forsciint.2019.06.024 31302415

[B105] Yonemitsu K. Sasao A. Mishima S. Ohtsu Y. Nishitani Y. (2016). A fatal poisoning case by intravenous injection of “bath salts” containing acetyl fentanyl and 4-methoxy PV8. Forensic Sci. Int. 267, e6–e9. 10.1016/j.forsciint.2016.08.025 27591912

[B106] Zawadzki M. Chłopaś-Konowałek A. Nowak K. Wachełko O. Szpot P. (2020b). Quantification of 5F-CUMYL-P7AICA in blood and urine from an authentic fatality associated with its consumption by UHPLC–MS/MS. Forensic Toxicol. 39, 240–247. 10.1007/s11419-020-00555-6

[B107] Zawadzki M. Nowak K. Szpot P. (2020a). Fatal intoxication with N-ethylpentylone: A case report and method for determining N-ethylpentylone in biological material. Forensic Toxicol. 38, 255–263. 10.1007/s11419-019-00483-0

[B108] Zawadzki M. Wachełko O. Chłopaś A. Paweł K. (2021). Quantification and distribution of 4 - fluoroisobutyryl fentanyl (4-FiBF) in postmortem biological samples using UHPLC – QqQ - MS/MS. Forensic Toxicol. 39, 451–463. 10.1007/s11419-021-00584-9

