# Supplementary material for: Fatal cases involving new psychoactive substances and trends in analytical techniques
Source: Front Toxicol. 2022 Oct 25;4:1033733. doi: 10.3389/ftox.2022.1033733 (PMC9640761; doi:10.3389/ftox.2022.1033733)
Supplement: Supplementary file 1 [file Table1.docx]

**Fatal cases involving new psychoactive substances and trends in analytical techniques**

Ettore Ferrari Júnior^1,2^, Bruno Henrique Monteiro Leite^1^, Eliude Barbosa Gomes^1^, Tales Mateus Vieira^3^, Pedro Sepulveda^4^, Eloisa Dutra Caldas^2*^

^1^ Forensic Analysis Laboratory, Criminalistics Institute, Civil Police of the Federal District, 70610-907, Brasília, Federal District, Brazil

^2^ Laboratory of Toxicology, Department of Pharmacy, University of Brasília, Campus Darcy Ribeiro, 70910-900, Brasília, Federal District, Brazil

^3^ Brainfarma Pharmaceutical Company, 75132-020, Anápolis, Goiás State, Brazil

^4^ Department of Pharmacy, University of Brasília, Campus Ceilândia, 72220-275, Brasília, Brasília, Federal District, Brazil

**SUPPLEMENTARY MATERIAL**

**Table S1** ǀ Analytical method details in fatal/postmortem cases analysis involving new psychoactive substances, from 2016 to 2021.

| **Reference** | **Analytical techniques** | **Extraction Method** | **LOD/LOQ**  **(ng/mL or ng/g)** ^1^ | **Substance (class)** | **Biological fluid/tissue, analyte concentration and number of postmortem/death cases** ^1^ | **Other substances detected (mainly NPS)** |
| --- | --- | --- | --- | --- | --- | --- |
| Adamowicz et al., 2016 ^2^ | LC-MS/MS | LLE | 0.036 / 1 | α-PVP (cath.) | Blood (1.1-6200); n = 12 | UR-144, BZDs, COC, THC, amphetamne, methadone, pentedrone, 3-MMC, ethcathinone |
| Beck et al., 2016 | LC-MS/MS: identification/quantification; LC-HR/MS: identification | PP | 0.2 / - | α-PVP (cath.) | Serum (62.6-304); n=2 | Opioids, BZDs, cannabis. Only α-PVP detected: 33% of the cases |
| Coopman et al., 2016 ^2^ | UPLC-MS/MS | LLE | 2.1 / 2.1 | Ocfentanil (opioid) | Blood (15.3); n=1 | Acetaminophen, caffeine |
| Fujita et al., 2016 | LC-MS/MS | QuEChERS | - | Mepirapim (SC); α-EAPP (opioid) | Blood: Mepirapim (950); α-EAPP (3100); n=1 | ND |
| Gieron and Adamowicz, 2016 ^2^ | LC-MS/MS | PP | 0.06 / 0.1 | AB-CHMINACA (SC) | Blood (1.5); blood from brain (2.2); blood from lung (2.7); blood from liver (0.3); blood from kidney (1.3); blood from intestines (1.0); urine (0.1); n=1 | Ethanol |
| Kristofic et al., 2016 | LC-QTOF: screening; LC-MS/MS: quantification | SPE | - | 25C-NBOMe (PEA) | 25C-NBOMe: blood (0.48-2.07), urine (1.73-27.43), brain (19.10), spleen (27.13), lung (25.21), liver (15.20), kidney (25.06); 2C-C: blood (0.12), urine (0.11-0.38); n=3 | Blood and urine: 25C-NBOMe, 25C-NBOH, 2C-C |
| Liveri et al., 2016 ^2^ | GC-MS | SPE | LOD: Blood/urine (0.002 -0.01) / LOQ: Blood (0.4-3); urine: (0.8-6) | MDPV and pentedrone (Cath) | MDPV: blood (46), urine (1300); pentedrone (mg/L): blood (160), urine (12000); n=1 | Blood and urine: Etizolam, ephedrine, olanzapine, mirtazapine |
| Papsun et al., 2016 ^2^ | LC-QTOF: screening; LC-MS/MS: quantification | LLE | 1 / - | MT-45 (Piperazine); Etizolam (D-BZD) | Blood: MT-45 (520); etizolam (35); n=1 | ND |
| Poklis et al., 2016 ^2^ | UPLC-MS/MS | SPE | - / 1 | Butyryl Fentanyl (opioid) | Butyryl fentanyl: P. blood (99-3.7), H. blood (220-9.2), VH (32-9.8), GSC (590-4000), brain (93-63), liver (41-39), bile (260-49), urine (64-2); n=2 | Acetyl fentanyl, alprazolam |
| Rojkiewicz et al., 2016 | HPLC-MS and GC-MS | LLE | 7 / 12 | 4-FBF (opioid) | Blood (91-112), urine (200-414), liver (902-411), kidney (136-197); n=2 | ND |
| Shanks and Behonick, 2016 ^2^ | LC-MS/MS | LLE | 0.1 / 0.2 | 5F-AMB (SC) | Blood (0.3); n=1 | ND |
| Yonemitsu et al., 2016 ^2^ | LC-MS/MS and GC-MS: screening; LC-MS/MS: quantification | QuEChERS | - | Acetyl fentanyl (opioid); 4-MeO-PV8 (Cath) | Acetyl fentanyl: F. blood (153), urine (240), GSC (880); 4-methoxy PV8: F. blood (389), urine (245), GSC (500); n=1 | 7-aminonitrazepam, phenobarbital, methylphenidate, chlorpromazine, risperidone |
| Angerer et al., 2017 ^2^ | GC-MS, HPLC-MS/MS and HPLC-PDA: screening; LC-MS/MS: quantification | LLE | 0.01-0.03 / 0.1-0.25 | 5F-PB-22, AB-CHMINACA and 5F-ADB (SC) | F. blood: 5F-PB-22 (0.37), n=1, AB-CHMINACA (4.1), n=1; 5F-ADB (0.38), n=1 | Metabolites of 5F-ADB, NE-CHMIMO and MDMB-CHMICA; olanzapine, trimipramine |
| Bottinelli et al., 2017 ^2^ | GC-MS, LC-DAD: screening; GC-MS/MS: quantification | SPE | - / 50 | 3-MMC (Cath) | 3-MMC: P. blood (249), CAR (609), VH (2988), bile (1291), urine (29694); n=1 | ND |
| Dwyer et. Al. 2017 | GC-MS: screening; LC-MS/MS: quantification | LLE/SPE | - | Fentanyl and acetylfentanyl (opioid) | Blood: acetylfentanyl (0.13–2100); fentanyl (0.24-74.3); urine: only qualitative; n=41 | Ethylone, ketamine, BZDs, COC, heroin and other opioids |
| Ellefsen et al., 2017 ^2^ | LC-MS/MS and GC-MS | LLE | - / 0.001 | 3-FPM (PHEN); U-47700 (opioid) | 3-FPM: P. blood (2400), aortic blood (600); U-47700: P. blood (360); n=1 | Amitriptyline, nortriptyline, methamphetamine, amphetamine, Flubromazolam, delorazepam and others BZD |
| Guerrieri et al., 2017 ^2^ | LC-MS/MS | LLE-LTP | - | Acrylfentanyl (opioid) | Blood (0.01-5); n=40 | 4-MeO-α-POP, MO CHMINACA, amphetamines, BZDs, 4Cl-α-PVP, N-etylnorhexedron, 4Cl-isobutylfentanyl, MDMA. THC |
| Johansson et al., 2017 ^2^ | LC-TOF-MS: screening; LC-MS/MS: quantification | LLE | - / 0.01 | 3-MeO-PCP (PCY) | Blood (50 to 180); (n=6); blood (380 μg/g) in a mono-intoxication case; n=1 | Buprenorphine, 5-MeO-MIPT, fentanyl, tramadol |
| Krotulski et al., 2017 | LC-QTOF: screening and metabolite investigation; LC-MS/MS: quantification | SPE | - | THFF and U-49900 (opioid); MeO-PCP (PCY) | Blood and urine, respectively: THFF (339; >5000); U-49900 (1.5; 2.2); MeO-PCP (1.0; 31.8); n=1 | alprazolam, paroxetine, topiramate, zolpidem, trazodone, aripiprazole, chlorpheniramine, dextro/levomethorphan, promethazine |
| Paul et al., 2017 | LC-MS/MS | NA | 0.01-2.0 / 0.1-2.0 | AB-CHMINACA, UR-144, XLR-11 and JWH-022 (SC) | Blood: AB-CHMINACA (8.2), n=1; UR-144 (12.3), XLR-11 (1.3) and JWH-022 (3), n=1 | ND |
| Potocka-banas et al., 2017 | LC-MS/MS | LLE | 1 / 5 | α-PVP (Cath) | α-PVP: blood (174), urine (401), brain (92), liver (190), kidney (122), GSC (606); n=1 | Midazolam, metoclopramide |
| Rojek et al., 2017 ^2^ | LC-MS/MS | LLE | - / 0.05-10 | UR-144 (SC); Pentedrone (Cath) | Blood: UR-144 (2.1), n=1; UR-144 (1.4), pentedrone (2300), (n=1); UR-144 (4), pentedrone (290), n=1 | ND |
| Staeheli et al., 2017 ^2^ | LC-MS/MS | LLE | - | MDAI (AI); 2-MAPB (Cath) | P. blood: MDAI (38); 2-MAPB (21); n=1 | diphenhydramine, morphine |
| Wiergowski et al., 2017 ^2^ | HPLC-QTOF-MS: screening; UPLC-MS/MS: quantification | PP/LLE | 0.0053-0.0013 / 0.0159-4.0 | 25B-NBOMe (PEA); 4-CMC (Cath) | Blood. 25B-NBOMe (38.4-661), 4-CMC (0.887-2.14); n=2 | THC |
| Allibe et al., 2018 ^2^ | LC-MS/MS: identification/ and quantification; LC-HRMS (QTOF): metabolite investigation | SPE | 0.01/ 0.05 | Ocfentanil (opioid) | Ocfentanil: P. blood (3.7), CAR (3.9), VH (2.0), bile (8.4) , GSC (2.5), nasal swabs (nd); n=1 | Caffeine, acetaminophen, heroin and other opioids |
| Atherton et al., 2018 | GC-MS | LLE | - / 10 | N‐ethylpentylone (Cath) | P. blood (31-953); n=4 | Fentanyl, COC, hydrocodone, alprazolam |
| Ballesteros et al., 2018^2^ | LC-MS/MS and GC-MS: detection | SPE | 20 / - | 4-MEC and α-PVP (Cath) | α-PVP: blood (9-1200); urine: detected 4-MEC and α-PVP; n=2 | Amphetamine, MDMA, MDA, lormetazepam and other BZD, THC-COOH |
| Costa et al., 2018 ^2^ | LC-MS/MS | LLE | 1 and 5 / - | N‐ethylpentylone (Cath) | Blood (170); n=1 | ND |
| Fagiola et al., 2018 ^2^ | GC-MS or LC-MS: screening; LC-MS/MS | LLE | 2.5 (LC-MS/MS); 200 (GC–MS or LC-MS, for cathinones) / - | Mitragynine and 7-OH-mitragynine; Pentylone, methylone and butylone (Cath) | Blood/urine: Mitragynine, n=2; mitragynine and 7-hydroxymitragynine, n=3; pentylone, methylone and butylone, n=1 | Synthetic opioid |
| Gerace et al., 2018 ^2^ | UHPLC-MS/MS | LLE | 0.6 / 2 | U-47700 (opioid) | Blood (380); urine (10400); pubic hair (5700) n=1 | ND |
| Koch et al., 2018 ^2^ | LC-MS/MS | PP/LLE/ SPE | - / 1 | U-47700 (opioid) | Blood: 42 min (370), 9 h. (37), 24h. (6.3), 33 h. (2.1), 41 h. (2.3); urine (2); n=1 | Flubromazepam and other BZD, lidocaine, pregabalin |
| Krpo et al., 2018 | UHPLC-QTOF-MS: screening; UHPLC-MS/MS: confirmation and quantification | LLE | - | 5-APB (PEA) | P. blood (860); n=1 | Ethanol, THC |
| Kusano et al., 2018 ^3^ | LC-MS/MS: screening and quantification LC-QTOF-MS: screening | PP | 0.005-0.1 / - | Diphenidine (PCY); 5F-ADB (SC) | Blood: 5F-ADB (0.19 ± 0.04), diphenidine (12 ± 2.6); n=1 | ND |
| Lehmann et al., 2018 ^2^ | LC-MS/MS | SPE/  QuEChERS | 0.4-5 / - | Methoxetamine (PCY); 4‑MEC, MDPV and α‑PVP (Cath) | F. blood: 4-MEC (8 to 118), MDPV (3 to 396), MXE (2 to 385) and α-PVP (4); H. blood, P. fluid, bile, stomach content, brain, liver, lung, kidney, muscle, urine: 4-MEC (8 to 901), MDPV (3 to 1202), MXE (1 to 1391); n=2 | ND |
| Maher et al., 2018 | HPLC-DAD; LC-QTOF-MS: identification; LC-MS/MS: identification/ quantification | LLE | 0.05- 0.16 / - | Cyclopropylfentanyl and crotonylfentanyl (opioid) | F. blood: (16.6-28.9); n=4 | ND |
| Majchrzak et al., 2018^3^ | LC-MS/MS | LLE | Body fluids: 9.0-27.2; tissues: 15.0-46.0 / - | N‑PP (Cath) | N-PP: blood (3100), eyeball fluid (4400), liver (5900), kidney (5400), brain (2300); n=1 | ND |
| Mardal et al., 2018 ^2^ | UHPLC-MS/MS: identification and quantification; UHPLC-HR-MS/MS: metabolite investigation | LLE/  PP | - / 7- 68 | Methoxyacetylfentanyl (opioid) | F. blood (22), brain (74), n=1; F. blood (23), urine (120), n=1; F. blood (56), n=1 | Oxycodone |
| Moody et al., 2018 ^2^ | LC–MS/MS: quantification; LC-TOF: screening | SPE | 0.0125-0.25 / 0.05-0.5 | 4-ANPP, 2-Furanylfentanyl, carfentanil, fluorobutyrylfentanyl, U-47700, acrylfentanyl, butyrylfentanyl, fluorofentanyl, 4-methoxybutyrylfentanyl and valerylfentanyl (opioid) | Blood: 4-ANPP (0.1-410), n=1549; 2-furanylfentanyl (0.1-710), n=1228; carfentanil (0.1-120), n=697; fluorobutyrylfentanyl (0.1-760), n=563; U-47700 (0.2-3800), n= 543; acrylfentanyl (0.1-29), n=266; butyrylfentanyl (0.1-760), n=142; p-fluorofentanyl (0.1-1), n=31; o-fluorofentanyl (2.4), n=1; 4-methoxybutyrylfentanyl (79), n=1; valerylfentanyl (0.44), n=1 | ND |
| Nooble et al., 2018 ^2^ | LC-QTOF-MS: screening; UHPLC-MS/MS: quantification | PP/SPE | 1-5 / 5 | Fentanyl (opioid) | Blood: fentanyl (7–39); n=17 | ND |
| Partridge et al., 2018 ^2^ | LC-QTOF: screening, quantification and metabolite investigation | LLE | 0.8-3 / - | U-47700 (opioid); Diclazepam and flubromazepam (D-BZD) | P. blood: U-47700 (330), diclazepam (70), flubromazepam (10); n=1 | Methamphetamine, amphetamine, lorazepam, DOC |
| Pieprzyca et al., 2018 ^2^ | LC-MS/MS | PP | 5 / 10 | PV8 (Cath) | PV8: blood (70-260), urine (110 to 130), liver (20-40), kidney (10-40); n=2 | Clindamycine, paracetamol, metamizole, lidocaine, dextromethorphan, drotaverine |
| Rohrig et al., 20180 ^2^ | GC-MS: screening; GC-NPD: screening and quantification | SPE | 25 / - | U-47700 (opioid) | U-47700: H. blood (260), F. blood (400), VH (90), brain (380), liver (280), urine (4600); n=1 | THC |
| Strehmel et al., 2018 | LC-QTOF-MS: screening; LC-MS/MS: quantification | PP | - | U-47700 (opioid) | U-47700 (µg/ml): F. blood (290), H. blood (12500), liver (9900), urine (240), GSC (570), bile (2300), CSF (400); n=1 | Caffeine, nicotine, oxycodone, theobromine, theophylline |
| Tomczak et al., 2018 ^2^ | GC-MS | LLE | 0.3 / 1 | 4‑CMC (Cath) | Blood: (56.2-1870); n= 6 | Diazepam, MDMA, MDA, THC, amphetamine, 3-MMC, Estazolam, COC metabolites |
| Adamowicz et al., 2019 ^2^ | LC-MS/MS | PP | - / 0.1 | AMB-FUBINACA and EMB-FUBINACA (SC) | AMB-FUBINACA, EMB-FUBINACA, respectively: blood (ND, ND), urine (4.7, 0.2), urine hydrolyzed (8.2, 0.1), kidney tissue (0.2, 0.4), kidney (bloody fluid) (0.1, 0.1), liver tissue (0.2, 0.2), liver (bloody fluid) (0.8, ND), stomach tissue (0.9, 2.7), stomach content (5.8, 36.2), intestine tissue (0.8, 3.5), intestine (bloody fluid) (0.1, 0.2), lung tissue (ND, 1.4), lung (bloody fluid) (0.1, ND), brain (0.6, 0.6); n=1 | Lorazepam, haloperidol, lidocaine |
| Al-Matrouk et al., 2019 | LC–MS/MS and LC-HRMS: screening | SPE | - | 5F-AB-PINACA, AB-PINACA, AB-CHIMICA, FUB-AMB, 5F-AB-PINACA, 5F-AKB-48, 5Cl-AKB-48, ADB-PINACA and 5F-ADB (SC) | Urine: only qualitative analysis (n=6) | ND |
| Ameline et al., 2019 ^2^ | GC-MS: screening; UPLC-MS/MS: quantification | LLE | - | 3-MeO-PCP (PCY) | P. blood (498), CAR (743), urine (16.7), hair (15600); n=1 | ND |
| Chesser et al., 2019 ^2^ | LC-MS/MS | SPE | 0.05-0.1/0.1 | 4-ANPP, acetylfentanyl, fentanyl, furanylfentanyl, norfentanyl and U-47700 (opioid) | Blood (femoral, cardiac, iliac, subclavian) (0.1 - 45; 0.1-227; 0.1-98; 0.2-89; 0.1-38; 0.4->500); VH (0.1-28; 0.1-45; 0.2-68; 0.3-14; 0.1-19; 0.1-328); brain (ND; 0.1->600; 0.3-176; 0.4-167; 0.4-22; 1->600), for 4-ANPP, acetylfentanyl, fentanyl, furanylfentanyl, norfentanyl, U-47700, respectively; n=58 | ND |
| De Jong et al., 2019 | UPLC-MS/MS: quantification; LC-QTOF-MS: screening | SPE | - | 3-MeO-PCP (PCY) | Serum (123), blood (152); n=1 | amphetamine |
| Deville et al., 2019 ^2^ | GC-MS and UPLC-TOF-MS: screening and identification; HPLC-DAD: quantification | LLE | - | MDAI (AI); 5-EAPB (Cath) | MDAI, 5-EAPB, 5-MAPB, 5-APB, respectively: blood (2090, 6450, 89, 546); urine (69400, 14800, 1000, 48800); n=1 | Oxazepam |
| Fagiola et al., 2019 ^2^ | LC-MS/MS | LLE | 2.5 / - | Cyclopropylfentanyl (opioid) | CAR (5.6-82); n=5 | BZD, COC, opioids, methamphetamine, despropionyl fentanyl, THC-COOOH |
| Fels et al., 2019 ^2^ | LC-QTOF-MS: identification and quantification | LLE/SPE | 5 / 10 | U-47700 (opioid) | U-47700: F. blood (27–2200), H. blood (39–4900), liver (72–8400), urine (100-5400), VH (14-11000), P. fluid (43-4600), GSC (630-180000), putrefaction fluid (61-320); n=26 | Fentanyl and analogs, amphetamine, methamphetamine, MDMA, opioids, flubromazepam and others BZD, N-Ethylpentylone and others cathinones, 3-MeO-PCP and others phencyclidine analogs, SCs, 3-FPM, MDAI, mitragynine |
| Freni et al., 2019 ^2^ | LC-MS/MS | SPE | 0.03-0.1 / - | Furanylfentanyl and 4-ANPP (opioid) | Furanyl fentanyl and 4-ANPP, respectively: CAR (11.8± 0.7; 93.5 ± 7.6), F. blood (2.7± 0.1; 50.4 ± 2.9), urine (71.3 ±3.3; 171.7 ± 13.8), bile (7.7 ± 0.8; 41.9 ± 1.6), CSF (2.6± 0.2; 10.2 ± 0.6), GSC (40.1 ± 11.2; 24.2 ± 2.0); n=1 | ND |
| Gaulier et al., 2019 ^2^ | LC-QTOF: screening; LC-MS/MS: quantification | SPE | 0.05/ 0.1 | Carfentanil (opioid) | Blood (4.20), urine (0.40); n=1 | Diclazepam and others BZD, heroin and others opioids, COC, MDMA, benzoylfentanyl and 4-fluobutyrylfentanyl, ethylhexedrone, AB-FUBINACA, MAM 2201, methoxetamine |
| Ivanov et al., 2019 | GC-MS: detection; HPLC-UV: quantification | LLE | 5F-ADB  25 / - | 5F-ADB and FUB-AMB (SC) | 5F-ADB: blood (3.7); n=1 | ND |
| Kovács et al., 2019 ^2^ | LC-MS/MS | LLE | 0.01-10 / - | N-ethylhexedrone (Cath); ADB-FUBINACA (SC) | Blood: NEH (285), ADB-FUBINACA (0.08); n=1 | THC, THC-COOH |
| Kriikku et al., 2019 ^2^ | UPLC-TOF-MS: screening; GC-MS: quantification | SPE | 10 / 20 | U-47700 (opioid) | Blood (150–2000), n=10; urine (20-2200), n=12 | m-CPP, phenazepam and others BZD, buprenorphine, pregabalin, THC, α-PVP, amphetamine |
| Krotulski et al., 2019 | LC-QTOF: qualitative analyses and metabolite identification | LLE/  SPE | - | 4F-MDMB-BINACA (SC) | Blood and urine: qualitative analysis; n = 20 | 5F-MDMB-PINACA (5F-ADB). 4F-MDMB-BINACA |
| Lehmann et al., 2019 | LC-MS/MS | SPE/  QuEChERS | - | Diclazepam and pyrazolam (D-BZD); 3-FPM (PHEN) | Diclazepam, pyrazolam, 3-FPM, respectively: F. blood (1; 28; 10), H. blood (1; 28; 9), urine (1; 500; 120), P. fluid (1; 11; 16), CSF (4; 45; 13), bile (17; 340; 190), brain (23; 100; 76), liver (34; 92; 160), lung (21; 98; 89), kidney (45; 160; 94), muscle (19; 88; 56), stomach contents (16; 380; 84); n=1 | 2-FA, 2-FMA, methiopropamine, amphetamine, caffeine, lorazepam |
| Margasińska-Olejak et al., 2019 ^2^ | LC-MS | LLE | - | 3-MMC (Cath) | Blood (800), VH (153), GSC (5.5 mg); n=1 |  |
| Nash et al., 2019 ^2^ | LC-QTOF: screening and quantification | LLE | - | Furanylfentanyl (opioid); MMMP (Cath) | P. blood: furanylfentanyl (1.6), MMMP (6.7); n=1 | THC, mirtazapine, paliperidone, quetiapine, 4-ANPP |
| Theofel et al., 2019 ^2^ | LC-MS/MS: quantification | PP/SPE | 3 / 5 | N-ethyldeschloroketamine (PCY) | N-ethyldeschloroketamine: liver (6137), urine (3468), bile fluid (3290), GSC (3086), H. blood (2159), liquor (1564), F. blood (375); n=1 | Deschloroketamine, metamizole, opioids, ibuprofen, venlafaxine |
| Yeter and Erol Öztürk, 2019 ^2^ | LC - HRMS: identification and quantification | SPE | Blood: 0.08; urine: 0.10 / blood: 0.10; urine: 0.12 | 5F-ADB and its methyl ester hydrolysis metabolite (SC) | Blood: 5F-ADB (0.10-1.55), 5F-ADB metabolite (0.15-23.4), n=70; urine: 5F-ADB metabolite (0.28-72.2), n=34. | AMB-FUBINACA, ADB-FUBINACA, 5F-MDMB-PICA, JWH-018, MAB-CHMINACA, AB-CHMINACA, CUMYL-4CN-BINACA, cannabis, MDMA, COC, heroin, amphetamine, methamphetamine |
| Adamowicz et al., 2020a | LC-MS/MS | LLE | 0.3 / 5 | α-PiHP (Cath) | α-PiHP: blood (69), urine (2072) and bile (341), solid tissues (7–478); n=1 | 4-CMC, N-ethylhexedrone, BZE, MDMA |
| Adamowicz et al., 2020b ^2^ | LC-MS/MS | LLE | 0.01-0.20 / - | Benzylfentanyl (opioid) | Blood: Benzylfentanyl (66; 110); fentanyl (31; 32); norfentanyl (22; 41);4-FiBF (74); despropionyl-4-FF (6.5); n=3 | α-PHP, N-Ethylhexedrone, 5-APB (or 6-APB), 4-FMA, 4-FA,(α-PiHP), THC-COOH |
| Benedicte et al., 2020 ^b^ | GC-MS: screening; LC-HRMS: confirmation and metabolite identification | LLE | 0.5 / 1 | MPHP and N‐ethyl‐4′methylpentedrone (Cath) | MPHP and 4-MEAP, respectively: F.blood (47; 1.6), CAR (97; 3.5), urine (2380; 49700); n=1 | THC, 4′-carboxi-PHP |
| Ditrana et al., 2020 ^2^ | HPLC-MS/MS | PP | Blood: 0.03-0.35; urine: 0.02-0.25 / blood: 0.08-1; urine: 0.06-0.5 | Cyclopropylfentanyl, methoxyacetylfentanyl, furanylfentanyl, acetylfentanyl, 4-ANPP and fentanyl (opioid) | Blood (0.2-9); urine (0.2-8900), for fentanyl derivatives; n=41 | Opioids |
| Garneau et al., 2020 | GC-MS: screening; LC-MS/MS: screening and quantification | SPE | - | 4-ANPP, furanylfentanyl, U-47700, p-fluorobutyrylfentanyl, methoxyacetylfentanyl, cyclopropylfentanyl/ crotonylfentanyl, acetylfentanyl, despropionyl fluorofentanyl and N-methyl U-47931 E (opioid) | Cardiac and F. blood, respectively: 4-ANPP (33-32; 18), furanylfentanyl (14-2.4; 0.89) and U-47700 (54-45; 26); n=2. Cardiac and F. blood, respectively: 4-ANPP (5.1; 9.7), p-fluorobutyrylfentanyl (31; 27), methoxyacetylfentanyl (70; 14), cyclopropylfentanyl/ crotonylfentanyl (0.15; 0.1), only detected: U-47700, acetylfentanyl, despropionyl fluorofentanyl, N-methyl U-47931 E; n=1 | Amphetamine, metamphetamine, COC, methadone, THC, BZDs |
| Hvozdovich et al., 2020 | LC-MS/MS | SPE | - | 5F-ADB, FUB-AMB, 5F-AMB, MDMB-FUBINACA, and AB-CHMINACA (SC) | Blood and/or urine: only qualitative analysis; n=54. 5F-ADB was the most prevalent substance | Ketamine, morphine, and others |
| Kriikku et al., 2020 ^2^ | GC-NCI-MS | LLE | 1 / - | Flualprazolam (D-BZD) | Blood (3.0-68); n=33 | ND |
| Krotulski et al., 2020a | LC-QTOF-MS: screening and metabolite investigation | LLE/SPE | - | APP‐BINACA (SC) | Blood and urine: only qualitative analysis; n=11 | 4F‐MDMB‐BINACA, 5F‐MDMB‐PICA, 5F‐MDMB‐PINACA, fentanyl, etizolam, THC, opioids |
| Krotulski et al., 2020b ^3^ | LC-MS/MS: quantification; LC-QTOF-MS: metabolite investigation | LLE | <0.02 / - | Isotonitazene (opioid) | Blood (0.4-9.5), n=18; urine (0.6-4.0), n=6; VH (0.1), n=1 | 4-ANPP, and U-47700, etizolam, COC |
| Lehmann et al., 2020 ^2^ | LC-MS/MS | SPE | 0.4–4 / 5 | PMMA, PMA, PMEA, 2-FA, 4-FA, 2-FMA, 3-FPM, 2-DPMP, MDEA, MDMA, MDA and methiopropamine (PEA); 3-MeO-PCP and MXE (PCY); m-CPP (piperazine); MDPBP, MDPV, 4-MEC, methedrone, methylone and α‑PVP (Cath); U-47700 (opioid); pyrazolam, diclazepam; delorazepam; lormetazepam (D-BZD) | Amphetamine and analogs (PMMA, PMA, PMEA, 4-FA, 2-FA, 2-FMA, methiopropamine, MDMA, MDA, MDEA, amphetamine, n=13): 4.5-185000 (urine); 2.2-2500 (blood). M-CPP (n=1): 130 (urine), 5.3 (blood); MXE (n=4): 6.6-22300 (urine), 1-390 (blood), 810 (kidney); 3-FPM (n=1): 120 (urine), 5.3 (blood); U-47700 (n=1): 1500 (urine); 2-DPMP (n=1): 52 (urine), 5.2 (blood); 3-MeO-PCE (n=1): 3.6 (urine); Synthetic benzodazepines (Pyrazolam, diclazepam, delorazepam, lormetazepam, n=1): 1-100 (blood); Cathinones (4-MEC, MDPV, methedrone, methylone, MDPBP, α‑PVP, n=4: 6.2-830 (urine). 3.6-340 (blood), 53-230 (kidney); n=17 | Femoral blood and urine: 2‑DPMP, MXE, 3‑MeO‑PCE, PMMA, PMA, PMEA, methylone, metamphetamine,amphetamine, MDMA, methedrone, MDEA,4-MEC, methadone, EDDP, 2‑FA, 2‑FMA, MDPV, 3‑FPM, U-47700 |
| Tiemensma et al., 2020 | GC-MS and LC-MS | NA | - | Cumyl-PEGACLONE (SC) | Blood (0.73-3.0); n=5 | 5F-Cumyl-P7AICA, 5F-Cumyl-PEGACLONE, lignocaine, paliperidone, THC |
| Woods,  2020 ^2^ | GC-MS | LLE | <10 / 50 | Mebroqualone (Meth) | F. blood (10228; 115); n=2 | Lorazepam, oxycodone, diphenhydramine, amphetamine, methamphetamine |
| Zawadzki et al., 2020a ^2^ | UHPLC-MS/MS | LLE | - / 0.1 | 5F-CUMYL-P7AICA (SC) | Blood (2.8), urine (3.1); n=1 | ND |
| Zawadzki et al., 2020b ^2^ | UHPLC-MS/MS | LLE | - / 1 | N‐ethylpentylone (Cath) | P. blood (10600), urine (17600); n=1 | Eutylone |
| Arbouche et al., 2021 | LC-MS/MS: quantification; LC-HRMS: confirmation and metabolite investigation | LLE | - | 3-MeO-PCP (PCY) | F. blood (525), urine (384); n=1 | Methadone, THC |
| Brahan et al., 2021 ^2^ | GC-MS/MS | LLE | - / 1000 | 4-MEC (Cath) | 4-MEC: P. blood (14600), CAR (43400), urine (619000), VH right and left (2900, 4400), bile (43500), GSC (28200); n=1 | Hydroxyzine |
| Castellino et al., 2021 | GC-MS | LLE | 1.0 / - | Cyclopropylfentanyl (opioid) | Blood (14), n=1; Other case: only detected, n=1 | alcohol, COC, oxycodone |
| Cartiser et al., 2021 ^2^ | GC-MS | SPE | - | 4-MPD (Cath) | 4-MPD: P. blood (1285),  CAR (1128), urine (>10,000), bile (1187), VH left and right (734;  875); n=1 | COC, sildenafil, bromazepam, nevirapine |
| Chan et al., 2021 ^2^ | LC-MS/MS | PP | - | Carfentanil (opioid) | P. blood (0.5), (n=1); iliac blood (0.9), n=1 | Naproxen, desloratadine, olopatadine, zolpidem |
| Ferrari Jr. and Caldas, 2021 ^2^ | UHPLC-MS/MS | QuEChERS | 4 / 10 | N-ethylpentylone (Cath) | Blood (597); n=1 | ND |
| Gicquel et al., 2021 ^2^ | LC-MS/MS: screening; LC-HRMS (QTOF): screening and quantification | SPE | 5 / 10 | 2F-DCK and 3-MeO-PCE (PCY) | 2F-DCK, 3-MeO-PCE and 5-MeO-DMT, respectively: P. blood (1780; 90; 52), urine (6100; 6300; 2200), bile (1200; 3500; 1700), VH (1500; 66; 155); n=1 | Amphetamine, COC, THC, levamisole, lorazepam |
| Hofmann et al., 2021 ^2^ | HPLC-MS/MS | PP | 1.8-2.6 / 4.6-6 | 5-APB and 6-APB (PEA) | 5-APB and 6-APB, respectively: C. blood (2400; 660), P. blood (850; 300), urine (8700; 3400), stomach content (65000; 4500), bile (4700; 2100), muscle (1400; 370), brain (7700; 1700), kidney (380; 64), liver (6900; 1600), lung (3300; 760); n=1 | ND |
| Kronstrand et al., 2021^2^ | LC-MS/MS: quantification. LC-QTOF-MS: metabolite investigation | PP | - / 2 | Methoxyacetylfentanyl (opioid) | F. blood: (18-140); n=10 | Opioids, BZDs |
| Krotulski et al., 2021a | LC-MS/MS: quantification; LC-TOF-MS: screening; LC-QTOF-MS: metabolite investigation | LLE and PP/SPE | - / 1 | Eutylone (Cath) | Blood (1,2-11000), n=67; urine (60; 3400; and >10000), n=3; brain (6.2), n=1; liver (10000), n=1. | Blood: Fentanyl, MDMA, methamphetamine, etizolam and pther benzodiazpines, ketamine, COC, opioids, THC and prescribed medicines; urine: 5F-MDMB- PICA 3,3- dimethylbutanoic acid |
| Krotulski et al., 2021b ^3^ | LC-MS/MS: quantification; LC-QTOF-MS: metabolite investigation | LLE | <0.1 / - | Brorfine (opioid) | Blood: 0.1-10; n=20 | Opioids, BZDs, gabapentin, THC, cyclobenzaprine, COC |
| Krotulski et al., 2021c ^2^ | LC-MS/MS: quantification; LC-QTOF-MS: screening and metabolite investigation | LLE | 0.1 / 0.5 | Metonitazene (opioid) | Blood (0.5-33), urine (0.6-46); n=20 | Fentanyl, BZDs, opioids, amphetamine, metamphetamine, THC |
| Krotulski et al., 2021d | LC-QTOF-MS: identification and metabolite investigation | LLE/SPE | - | MDMB-4en-PINACA, 5F-MDMB-PICA and 4F-MDMB-BINACA (SC) | Blood: qualitative analysis; n=16 | COC metabolites, phenytoin, amphetamine, methamphetamine, opioids, THC |
| Mochizuki et al., 2021 ^2^ | LC-LIT-MS: detection and quantification; GC-MS: identification | SPE | 0.1-1 / - | 4-FMC, 4-MeO-α-PVP, 4-F-α-PVP and PV8 (Cath) | 4-FMC, 4-MeO-α-PVP, 4-F-α-PVP and PV8, respectively: H. blood (365; 449; 145; 218), F. blood (397; 383; 127; 167); n=1 | ND |
| Mueller et al., 2021 ^2^ | UHPLC-MS/MS | SPE | 0.01 / 0.05 | Isotonitazene (opioid) | Isotonitazene: F. blood (2.28; 0.59; 0.74), CAR (1.7; 1.13; 0.7), urine (1.88; 3.37; 0.19), humor vitreous (0.36; 0.12; 0.65), pericardiac fluid (6.7; 5.01; 2.66), lung (0.52; 17.9; 2.39), liver (0.04; 0.04; 0.02), kidney (1.61; 1.02; 0.67), heart (7.74; 2.17; ND), brain (18.6; 2.72; 4.45), spleen (4.4; 3.44; 2.62), muscle (1.15; 2.08; 1.0), CSF (ND; 0.88; ND), hair (75; 182; 32/35); n=3 | ND |
| Palazzoli et al., 2021 ^2^ | LC-MS/MS | PP/SPE | 0.1-0.5 / 0.5-1 | Mephedrone, DHM and NORMEP (Cath) | Mephedrone, NORMEP and DHM, respectively: F. blood: (1088; 47.1; 15.5), C. blood (1632; 50.2; 49.2), urine (4443; 740.2; 171.9), right lung (1808; 10.1; 15.4), left lung (1368; 29.6; 40.6), brain (1596; 15.6; ND), liver (1080; 9.5; 169.2), kidney (1468; 15.2; 39.2), bile (752; ND; ND); n=1 | COC |
| Solbeck et al., 2021 ^2^ | LC-MS/MS: quantification. LC-QTOF-MS, GC-NPD and GC-MS: screening. | SPE | 0.05 / 0.1 | Carfentanil (opioid) | Blood (< 0.1-9.2); n = 160 | COC, fentanyl, acetaminophen, BZD, metamphetamine, amphetamine, opioids |
| Theofel et al., 2021 ^2^ | GC-MS and LC-QTOF-MS/MS: screening; LC-MS/MS: quantification | NA | - | 2‐MAPB (Cath) | 2-MAPB: urine (167000), GSC (98900), bile (30800), liver (22200), H. blood (16700), F. blood (7300); n=1 | N-demethyl-2-MAPB and hydroxy-2-MAPB, diazepam, fephedrone, 2C-B, THC |
| Zawadzki et al., 2021 ^2^ | UHPLC-MS/MS | LLE | 0.05 / 0.1 | 4‑FiBF (opioid) | 4‑FiBF: blood (76.1- 257), urine (289-1000), VH (89.9-150), bile (1100-5410), brain (94.5-176), kidney (388-811), liver (1400-2040), stomach wall (1900) and GSC (2280-3990); n=4 | N-ethylpentylone, 4-CMC, α-PiHP, amphetamine, tramadol |

^1^ when necessary, concentrations reported in the studies were converted to ng/mL or ng/g to facilitate the comparison among the methods; ^2^ papers that described validation procedures; ^3^ papers that described quantitation by standard addition; C-NMR: carbon-13 nuclear magnetic resonance; EI: electron impact ionization; ELISA: enzyme-linked immunoassay; FT-IR: Fourier-transform infrared spectroscopy; GC-IR: gas chromatography – infrared spectroscopy; GC-MS: gas chromatography coupled to mass spectrometry; GC-MS/MS: gas chromatography coupled to tandem mass spectrometry; GC-NCI-MS: gas-chromatography negative-chemical-ionization mass spectrometry; H-NMR: proton nuclear magnetic resonance; HPLC-DAD: high performance liquid chromatography-diode-array detector; HPLC–DAD-FLD: high performance liquid chromatography-diode-array and fluorescence detectors; HPLC-MS/MS: high performance liquid chromatography- tandem mass spectrometry; HPLC-UV: high performance liquid chromatography-ultraviolet detector; HRMS: high-resolution mass spectrometry; LC-DAD: liquid chromatography-diode-array detector; LC-HRMS: liquid chromatography-high-resolution mass spectrometry; LC-MS: liquid chromatography- mass spectrometry; LC-PDA: liquid-chromatography-photodiode array detector; LC-MS/MS: liquid chromatography- tandem mass spectrometry; LC-QTOF-MS: liquid chromatography-quadrupole time-of-flight mass spectrometry; LC–TOF-MS: liquid chromatography-time of flight mass spectrometry: LC-UV: liquid chromatography-ultraviolet detector; MRM: multiple reaction monitoring; NMR: nuclear magnetic resonance; NPS: new psychoactive substance; SIM: selective ion monitoring; UHPLC-MS/MS: ultra high performance liquid chromatography- tandem mass spectrometry; UHPLC-QTOF-MS: ultra high performance liquid chromatography- quadrupole time-of-flight mass spectrometry; UPLC-MS/MS: ultra performance liquid chromatography- tandem mass spectrometry; UPLC-TOF-MS: ultra performance liquid chromatography-time-of-flight mass spectrometry; UPLC-PDA: ultra performance liquid-chromatography-photodiode array detector; UV-VIS: ultraviolet/visible spectrophotometry. **Extraction methods**: LLE: liquid-liquid extraction; LLE-LTP: liquid-liquid extraction with low-temperature partition; PP: protein precipitation; QuEChERS: quick, easy, cheap, effective, rugged, and safe; SPE: solid phase extraction. **Substances**: 2-FA: 2-Fluoroamphetamine; 2-FMA: 2-Fluoromethamphetamine; 2-Oxo-PCE: N-ethyldeschloroketamine; 3‑FPM: 3-fluoro-phenmetrazine; 3-MMC: 3-methylmethcathinone; 4-FA: 4-Fluoroamphetamine; 4-FMA: 4-Fluoromethamphetamine; 4-FBF: 4-fluorobutyrfentanyl; 4‑FiBF: 4‑fluoroisobutyryl fentanyl; 4-MEAP: N‐ethyl‐4′methylpentedrone; 4-MEC: 4-methylethcathinone; 4-MPD: 4-methylpentedrone; 5F-MDMB-PINACA: 5F-ADB; α-PiHP: alpha-Pyrrolidinoisohexaphenone; AI: aminoindane; AMP: amphetamine; BZD: benzodiazepine; BZE: benzoylecgonine; Cath: synthetic cathinone; COC: cocaine; D-BZD: designer-benzodiazepine; DHM: dihydro-mephedrone; MDA: Methylenedioxyamphetamine; MDMA: Methylenedioxymethamphetamine; Meth: Methaqualone analog; MMMP: 2-methyl-4’-(methylthio)-2-morpholinopropiophenone; MAMP: metamphetamine; N‑PP: α‑propylaminopentiophenone; NA: not available; ND: non-detected; NORMEP; Nor-mephedrone; PCY: phencyclidine analog; PEA: phenethylamine; PHEN: phenmetrazine analog; PMMA: para-methoxymethamphetamine; SC: synthetic cannabinoid; THC-COOH: 11-Nor-9-carboxy-THC; THC: tetrahydrocannabinol; THFF: Tetrahydrofuranylfentanyl. **Biological fluid/tissues:** GSC: gastric content; C. blood: central blood; CAR: cardiac blood; P. blood: peripheral blood; F. blood: femoral blood; H. blood: heart blood; P. fluid: pericardial fluid; VH: vitreous humor; CSF: cerebrospinal fluid.
